# Global patterns and trends in cancer-related premature death and their impact on life expectancy across 185 countries: a population-based analysis

**DOI:** 10.1186/s40779-025-00645-9

**Published:** 2025-09-03

**Authors:** Ke-Xin Sun, Xin Liang, Qian Zhu, Hong-Liang Wu, Gong-Yi Zhang, Yi-Fei Yao, Xiang Li, Rong-Shou Zheng, Jing Zuo, Wen-Qiang Wei

**Affiliations:** 1 National Central Cancer Registry, National Cancer Center/National Clinical Research Center for Cancer/Cancer Hospital, Chinese Academy of Medical Sciences and Peking Union Medical College, Beijing, 100021 China; 2 Medical Statistics Office, National Cancer Center/National Clinical Research Center for Cancer/Cancer Hospital, Chinese Academy of Medical Sciences and Peking Union Medical College, Beijing, 100021 China; 3 Department of Anesthesiology, National Cancer Center/National Clinical Research Center for Cancer/Cancer Hospital, Chinese Academy of Medical Sciences and Peking Union Medical College, Beijing, 100021 China; 4 Department of Gynecologic Oncology, National Cancer Center/National Clinical Research Center for Cancer/Cancer Hospital, Chinese Academy of Medical Sciences and Peking Union Medical College, Beijing, 100021 China

**Keywords:** Cancer, Premature death, Death probability, Year of life lost (YLL), Potential gain in life expectancy (PGLE)

## Abstract

**Background:**

The level of premature deaths (deaths among those aged 30–69 years) caused by cancer is an important indicator of evaluating the level of cancer prevention and control. However, the current burden and temporal trends in cancer-related premature deaths, and their impact on life expectancy at the global, regional, and national levels are not clear.

**Methods:**

Cancer mortality data for 185 countries were obtained from the GLOBOCAN 2022 database. High-quality cancer mortality data and national population statistics for 47 countries were extracted from the United Nations and national cancer registry databases, covering the period 2003–2022. Countries were classified based on the human development index (HDI). The death probability, the year of life lost (YLL), and the potential gain in life expectancy (PGLE) attributable to premature deaths from site-specific and all-cancers combined were calculated.

**Results:**

Globally, the probability of premature cancer deaths was 6.49% (95% UI 6.49–6.50). The YLLs caused by cancer-related premature death were 163.86 million (95% UI 163.70–164.03), constituting 65.58% of the total cancer-related YLLs. The PGLEs were 1.16 years (95% UI 1.16–1.16). The premature death probability increased with higher HDI levels in men, but decreased in women. Cancer-related premature deaths as a proportion of total cancer deaths varied from 18.31% (95% UI 18.20–18.43) in Japan to 84.44% (95% UI 76.10–91.16) in São Tomé and Príncipe. Lung cancer was the leading cause of cancer-related premature deaths in men, and breast cancer ranked first in women. By eradicating premature deaths attributable to lung, liver, colorectal, and stomach cancer in men, and to breast, cervical, and lung cancer in women, 0.55 years (95% UI 0.55–0.55) and 0.49 years (95% UI 0.49–0.49) of PGLEs could be achieved, accounting for 48.67% and 42.24% of the total PGLEs, respectively. Cancer-related premature deaths decreased significantly in 38 countries during 2003–2022 (*P* < 0.05). The probability of premature cancer-related deaths decreased by more than 15.50% from 2015 to 2022 in 16 countries.

**Conclusions:**

Cancer-related premature deaths declined in many countries, with 16 of them having achieved the expected reduction by 2022. The current burden of cancer-related premature deaths is profound but varies around the world. Eliminating premature deaths from major cancer types could substantially increase life expectancy, underscoring the importance of prevention and treatment efforts for these cancers.

**Supplementary Information:**

The online version contains supplementary material available at 10.1186/s40779-025-00645-9.

## Background

While death in old age remains an inevitable aspect of life, premature mortality is defined within the global health context as deaths occurring before the age of 70 [1]. It represents a preventable challenge whose mitigation holds significant potential to enhance health equity and advance human well-being [2]. This is a key indicator for evaluating the effectiveness of national healthcare systems, providing insight into the potential for extending healthy life expectancy through targeted interventions [3]. The World Health Organization (WHO) has identified reducing premature mortality from non-communicable diseases (NCDs) as a critical objective under the sustainable development goals (SDGs), particularly SDG Target 3.4. This target emphasizes reducing about 1/3 of premature deaths from NCDs through prevention, treatment, and the promotion of mental health and well-being by 2030. Achieving this target requires a comprehensive understanding of the current patterns associated with premature deaths across geographic and socioeconomic areas. Previous research provided a global analysis of all-cause premature mortality [4]. Cancer is a major chronic disease that has also emerged as a prominent cause of premature deaths around the world [5], resulting in substantial economic losses for both families and society [6]. Consequently, research focusing on cancer-related premature mortality has the potential to provide deeper insights that can significantly enhance efforts aimed at preventing and controlling premature deaths.

Globally, cancer incidence and mortality rates continue to increase, driven by factors such as population aging, lifestyle changes, and environmental influences. According to GLOBOCAN 2022 estimates, there were approximately 20 million new cancer cases and 9.7 million cancer deaths in 2022, with an estimated 11.6 million new cases and 4.9 million deaths occurring within the population aged 30−69 years [7]. While previous reports have addressed the estimation of cancer-related premature mortality [8, 9], there remains a scarcity of studies that provide a comprehensive estimation of the current landscape of cancer-related premature mortality profiles and their impact on life expectancy using the most up-to-date global data.

In the present study, aimed at conducting a thorough assessment of the global burden of cancer-related premature mortality, we calculated a range of indicators, namely death probability, the year of life lost (YLL), and potential gain in life expectancy (PGLE), using the most recent data sourced from GLOBOCAN 2022, encompassing 185 countries and 39 cancer types. To examine temporal trends, we also utilized high-quality mortality data for 47 countries spanning 2003 to 2022. These results may shed light on how disparities in accessibility to cancer treatment, implementation of cancer prevention initiatives, and adoption of comprehensive public health policies across regions influence the overall burden of cancer-related premature mortality.

## Methods

### Data sources

The data sources for this study were described in detail in the Additional file 1: Data sources. Cancer mortality data for 2022, stratified by country, sex, age group, and cancer site (Additional file 1: Table S1), were extracted from the GLOBOCAN 2022 database (https://gco.iarc.who.int/today/en) [10]. This database, issued by the International Agency for Research on Cancer, uses the best available data sources to provide estimates of cancer incidence and mortality in 185 countries. The datasets for individual countries have undergone uniform quality control procedures, ensuring the validity and comparability of global cancer surveillance data.

For trend analysis, we obtained cancer mortality data (https://platform.who.int/mortality) [11] and national population statistics (https://population.un.org/wpp/) from the United Nations database [12], covering the period 2003−2022. Mortality data for China during the same period were retrieved from the cancer registry datasets of China. After quality control, datasets from 47 countries were included (Additional file 1: Data sources and Tables S2-S3).

The regional classification used in this study is from the United Nations. We obtained the country-level human development index (HDI) data from the United Nations Development Program (https://hdr.undp.org/) [13]. In the Human Development Report 2022, HDI was divided into 4 categories: very high HDI (HDI ≥ 0.80), high HDI (HDI 0.70−0.79), medium HDI (HDI 0.55−0.70), and low HDI (HDI < 0.55). All data included in this study were from secondary sources and did not involve human or animal case data, so ethical approval was not required.

### Indicators

YLL is the most common indicator used to quantify the impact of premature deaths. It is calculated as the loss of life years caused by specific deaths occurring before reaching the expected lifespan. It can also be conceptualized as a comparison between the expected lifetime of a group of individuals with a specific disease and the expected lifetime of a group of individuals without that disease [14].

To demonstrate the significant impact of premature deaths on the overall health of the population, we calculated the PGLE in a hypothetical scenario in which these premature deaths could be completely prevented [15]. PGLE is a crucial indicator in formulating public health policies because it underscores the benefit of effective prevention and control of specific diseases in prolonging the population’s life expectancy. It therefore provides vital insights to enable prioritization of public health interventions [16].

HDI is a composite statistic encompassing life expectancy, educational attainment, and per capita gross domestic product. It is frequently used in global comparative analyses to gauge socioeconomic progress across nations and regions [17]. We stratified countries using the HDI to analyze the association between development levels and cancer outcomes. This stratification offers valuable insights into the estimated mortality burden across regions of diverse socioeconomic development levels.

### Statistical analysis

The statistical analysis methods were described in detail in the Additional file 1: Methods for calculating indicators and Table S4. Cancer-related premature death was defined as death from cancer occurring between the ages of 30 and 69 years. The death count was recorded, and the age-standardized rate (ASR) was calculated using Segi’s world standard population. Corresponding indicators for cancer-related premature death, such as death probability, YLL, and PGLE, were calculated within this age range.

The probability of cancer-related premature death was calculated using mortality tables. The YLL was estimated by multiplying the estimated number of cancer deaths and the corresponding standard life expectancy at that age by the United Nations figure for life expectancy in Japan used as the standard population [12]. The ASR of YLL was calculated using Segi’s world standard population. We also calculated the proportion of YLL caused by cancer-related premature death as a proportion of total YLL from all cancer deaths.

We used PGLE as a key metric to evaluate hypothetical improvements in life expectancy if premature cancer deaths were eliminated. The PGLE was computed as the difference between the life expectancy after removing all cancer-related premature deaths and the baseline life expectancy. We also calculated the proportion of PGLE from cancer-related premature death relative to the total PGLE from all cancer deaths.

These indicators were analyzed by sex, region, country, and HDI level for 39 individual cancer sites and all cancers combined (International statistical classification of diseases and related health problems, 10th revision, code C00 to C97). The cancer classification was listed in Additional file 2: Table S1. The linear trend test of indicators across different HDI levels using Student’s *t*-test with a linear regression model. The 95% uncertainty interval (UI) was calculated using a bootstrap simulation method with replicated 1000 times for each stratum, assuming that all causes of death and cancer deaths in the population follow a Poisson distribution [18].

Joinpoint regression analysis was used to examine trends in the death probability, YLL, and PGLE attributable to premature cancer death from 2003 to 2022. The average annual percent change (AAPC) and 95% confidence interval (CI) were reported for each trend. We computed the reduction in probability of premature cancer-related death for 2022 compared with 2015 in each country by dividing the difference by the probability of 2015. If the 1/3 reduction target is to be achieved by 2030 with a consistent rate of decline, a 17.23% reduction should have been attained by 2022 [1]. To accommodate flexibility in evaluation, we set the expected reduction threshold at 90% of this value, equivalent to 15.50% [9].

Statistical analyses were performed using SAS v9.4 (SAS Institute Inc., Cary, NC, USA) and Joinpoint software v4.6.0.0 (National Cancer Institute, Bethesda, MD, USA). *P* < 0.05 (two-tailed) was considered statistically significant.

## Results

### Global distribution patterns in 2022

In 2022, 4847.00 thousand (95% UI 4842.52–4851.63) individuals died prematurely from cancer, accounting for 49.80% of global cancer-related deaths (Table 1). Overall, 2683.36 thousand (95% UI 2679.60–2686.20) cancer-related premature deaths (55.36%) occurred among men, and 2163.64 thousand (95% UI 2161.21–2166.91) (44.64%) among women. Globally, the probability of cancer-related premature death was 6.49% (95% UI 6.49–6.50), with 7.48% (95% UI 7.46–7.48) for men and 5.57% (95% UI 5.56–5.58) for women. Cancer-related premature death was responsible for 163.86 million (95% UI 163.70–164.03) YLLs, and 65.58% of the YLLs were attributed to cancer across all age groups globally. This consisted of 88.37 million (95% UI 88.26–88.46) YLLs in men and 75.49 million (95% UI 75.39–75.61) in women, 64.36% and 67.08% of the total cancer-related YLLs, respectively. The global PGLEs associated with eliminating cancer-related premature death were 1.16 years (95% UI 1.16–1.16). This was higher for women (PGLE = 1.16, 95% UI 1.16–1.17) than men (PGLE = 1.13, 95% UI 1.13–1.13) (Table 1; Additional file 2: Table S1).

**Table 1:** Cancer-related premature deaths (30 − 69 years) and YLLs by world region, sex, and HDI, 2022

Characteristics	Cancer-related premature deaths				YLLs caused by cancer-related premature deaths			Probability [% (95% UI)]	PGLEs [years (95% UI)]
	Numbers (thousand, 95% UI)	ASR (/10^5^, 95% UI)	Probability in all-ages^*^[% (95% UI)]	Probability of all deaths^^^ [% (95% UI)]	Numbers (million, 95% UI)	ASR (/10^5^, 95% UI)	Probability in all-ages^*^ [% (95% UI)]		
Both sexes									
World	4847.00 (4842.52−4851.63)	134.98 (134.85−135.11)	49.80 (49.77−49.84)	19.21 (19.19−19.23)	163.86 (163.70−164.03)	4573.88 (4569.43−4578.51)	65.58 (65.55−65.62)	6.49 (6.49−6.50)	1.16 (1.16−1.16)
Very high HDI	1348.28 (1346.11−1350.65)	134.71 (134.46−134.91)	37.01 (36.95−37.04)	29.01 (28.95−29.07)	42.91 (42.83−42.98)	4425.50 (4416.89−4432.18)	56.08 (56.02−56.13)	6.64 (6.63−6.66)	1.44 (1.44−1.44)
High HDI	2070.36 (2068.10−2072.49)	138.69 (138.51−138.83)	51.87 (51.82−51.92)	24.42 (24.39−24.45)	69.06 (68.98−69.13)	4659.41 (4653.70−4664.27)	67.39 (67.34−67.44)	6.71 (6.70−6.72)	1.28 (1.28−1.28)
Medium HDI	1063.98 (1062.35−1065.74)	124.64 (124.43−124.84)	68.47 (68.39−68.54)	12.34 (12.32−12.36)	38.07 (38.01−38.14)	4352.73 (4345.86−4359.92)	75.66 (75.57−75.72)	5.83 (5.82−5.84)	0.87 (0.87−0.87)
Low HDI	364.37 (363.19−365.40)	128.59 (128.15−128.94)	67.06 (66.97−67.18)	10.46 (10.42−10.49)	13.82 (13.77−13.86)	4586.12 (4570.40−4600.38)	67.25 (67.12−67.37)	5.93 (5.91−5.95)	0.76 (0.76−0.77)
Northern America	269.66 (268.66−270.66)	117.88 (117.44−118.35)	38.18 (38.07−38.28)	24.32 (24.21−24.41)	8.49 (8.46−8.53)	3873.17 (3859.55−3889.26)	56.82 (56.69−56.91)	5.84 (5.82−5.86)	1.26 (1.25−1.26)
Eastern Asia	1392.17 (1390.16−1394.83)	134.55 (134.36−134.83)	44.25 (44.21−44.32)	35.09 (35.03−35.16)	45.13 (45.07−45.23)	4449.17 (4443.09−4460.27)	62.38 (62.32−62.44)	6.60 (6.59−6.61)	1.45 (1.44−1.45)
Eastern Africa	157.22 (156.41−158.03)	152.10 (151.25−152.98)	66.42 (66.25−66.63)	11.61 (11.55−11.67)	6.03 (5.99−6.06)	5422.21 (5393.18−5447.92)	65.62 (65.39−65.87)	6.99 (6.95−7.04)	0.93 (0.93−0.94)
Middle Africa	47.97 (47.57−48.44)	122.14 (121.01−123.43)	65.41 (65.07−65.69)	8.38 (8.30−8.47)	1.81 (1.80−1.83)	4306.75 (4269.64−4350.20)	64.88 (64.51−65.20)	5.71 (5.65−5.78)	0.65 (0.65−0.66)
Northern Africa	125.74 (125.08−126.37)	140.70 (139.85−141.35)	61.52 (61.29−61.71)	19.10 (18.99−19.21)	4.47 (4.45−4.49)	4876.87 (4851.60−4904.56)	70.35 (70.11−70.58)	6.61 (6.57−6.65)	1.12 (1.11−1.12)
Southern Africa	42.04 (41.69−42.41)	171.94 (170.36−173.38)	60.16 (59.78−60.51)	9.25 (9.16−9.33)	1.52 (1.51−1.54)	5969.63 (5915.78−6018.02)	72.74 (72.37−73.05)	8.04 (7.97−8.12)	0.86 (0.85−0.87)
Western Africa	117.68 (117.05−118.35)	123.91 (123.27−124.72)	68.55 (68.34−68.79)	8.38 (8.34−8.43)	4.44 (4.42−4.46)	4377.49 (4354.73−4403.04)	69.35 (69.03−69.58)	5.78 (5.74−5.82)	0.63 (0.63−0.64)
Caribbean	28.51 (28.21−28.91)	140.04 (138.67−141.95)	44.21 (43.90−44.61)	20.52 (20.26−20.81)	0.96 (0.95−0.97)	4748.42 (4696.22−4810.03)	61.73 (61.32−62.16)	6.74 (6.68−6.83)	1.23 (1.22−1.25)
Central America	69.17 (68.64−69.75)	94.80 (94.13−95.60)	51.71 (51.48−51.96)	13.95 (13.85−14.07)	2.43 (2.41−2.45)	3266.31 (3235.37−3297.19)	63.73 (63.40−64.01)	4.56 (4.53−4.60)	0.85 (0.84−0.86)
South-Eastern Asia	459.60 (458.24−460.99)	149.79 (149.36−150.22)	64.18 (64.05−64.29)	18.87 (18.82−18.93)	16.07 (16.03−16.12)	5183.41 (5170.30−5197.33)	74.48 (74.34−74.57)	7.04 (7.02−7.06)	1.20 (1.20−1.21)
South-Central Asia	936.84 (934.66−938.93)	117.73 (117.48−117.99)	69.56 (69.48−69.62)	11.97 (11.94−12.00)	33.55 (33.46−33.63)	4120.14 (4109.53−4129.58)	76.33 (76.23−76.39)	5.50 (5.49−5.52)	0.83 (0.83−0.83)
Western Asia	144.23 (143.48−144.96)	138.22 (137.56−138.89)	56.90 (56.72−57.08)	24.80 (24.68−24.93)	4.97 (4.94−5.00)	4585.51 (4558.90−4610.23)	68.27 (68.06−68.45)	6.76 (6.73−6.80)	1.25 (1.25−1.26)
Eastern Europe	363.23 (362.06−364.54)	188.76 (188.15−189.41)	52.08 (51.99−52.19)	19.89 (19.83−19.98)	11.55 (11.51−11.58)	6248.92 (6227.22−6270.13)	68.10 (68.00−68.23)	9.09 (9.06−9.12)	1.41 (1.40−1.41)
Northern Europe	79.80 (79.10−80.39)	122.55 (121.50−123.51)	28.42 (28.21−28.61)	40.51 (40.18−40.88)	2.49 (2.47−2.51)	3944.81 (3912.84−3979.59)	46.60 (46.35−46.84)	6.18 (6.13−6.22)	1.43 (1.42−1.44)
Southern Europe	140.73 (140.11−141.49)	134.04 (133.40−134.76)	31.74 (31.63−31.88)	45.81 (45.55−46.06)	4.44 (4.42−4.47)	4366.88 (4341.15−4391.58)	51.22 (51.08−51.38)	6.65 (6.62−6.68)	1.59 (1.58−1.59)
Western Europe	181.40 (180.47−182.06)	137.50 (136.73−138.07)	32.08 (31.93−32.17)	43.25 (42.98−43.44)	5.67 (5.64−5.70)	4474.57 (4449.66−4491.88)	51.23 (51.08−51.34)	6.82 (6.78−6.84)	1.63 (1.62−1.64)
Australia & New Zealand	19.63 (19.33−19.88)	110.54 (108.80−112.17)	31.06 (30.72−31.47)	44.58 (43.74−45.44)	0.62 (0.61−0.63)	3613.14 (3549.19−3664.17)	50.23 (49.82−50.74)	5.52 (5.43−5.58)	1.39 (1.37−1.41)
Melanesia	6.26 (6.12−6.41)	175.62 (171.40−179.60)	69.19 (68.16−70.08)	16.77 (16.40−17.27)	0.23 (0.22−0.23)	6127.92 (5991.14−6267.32)	74.08 (73.03−75.10)	8.15 (7.96−8.35)	1.19 (1.17−1.22)
South America	264.60 (263.52−265.56)	128.83 (128.30−129.29)	48.19 (48.06−48.33)	18.86 (18.79−18.93)	8.97 (8.93−9.00)	4357.01 (4340.48−4374.19)	64.33 (64.18−64.48)	6.23 (6.20−6.25)	1.12 (1.12−1.13)
Micronesia & Polynesia	0.55 (0.50−0.60)	182.01 (166.17−196.95)	57.53 (54.46−59.56)	43.41 (38.99−47.51)	0.02 (0.02−0.02)	5894.85 (5384.74−6469.29)	72.92 (70.35−75.00)	8.89 (8.21−9.59)	2.00 (1.80−2.17)
Men									
World	2683.36 (2679.60−2686.20)	152.22 (152.00−152.38)	49.47 (49.43−49.50)	17.29 (17.27−17.31)	88.37 (88.26−88.46)	5000.78 (4994.05−5005.97)	64.36 (64.31−64.40)	7.48 (7.46−7.48)	1.13 (1.13−1.13)
Very high HDI	765.52 (763.69−767.12)	156.34 (155.98−156.68)	38.11 (38.03−38.16)	25.18 (25.11−25.24)	23.91 (23.86−23.96)	4991.92 (4981.35−5003.88)	56.18 (56.10−56.24)	7.84 (7.83−7.86)	1.45 (1.45−1.45)
High HDI	1244.17 (1241.91−1246.51)	169.26 (168.97−169.60)	52.15 (52.09−52.21)	22.86 (22.81−22.91)	40.62 (40.55−40.71)	5537.94 (5527.57−5550.51)	67.01 (66.95−67.07)	8.30 (8.28−8.31)	1.35 (1.35−1.36)
Medium HDI	529.91 (528.67−531.69)	125.40 (125.08 − 125.81)	66.86 (66.77−66.95)	10.43 (10.40−10.46)	18.54 (18.49−18.60)	4275.36 (4265.37−4289.17)	73.45 (73.35−73.54)	5.99 (5.97−6.01)	0.78 (0.78−0.78)
Low HDI	143.76 (142.91−144.60)	107.44 (106.90−108.03)	60.56 (60.38−60.81)	7.35 (7.31−7.40)	5.29 (5.26−5.33)	3699.54 (3677.09−3720.25)	59.56 (59.34−59.84)	5.15 (5.13−5.18)	0.55 (0.55−0.56)
Northern America	142.88 (142.18−143.64)	125.71 (125.09−126.44)	38.27 (38.12−38.45)	20.87 (20.76−20.98)	4.43 (4.41−4.45)	4034.37 (4012.41−4058.28)	56.15 (55.99−56.35)	6.34 (6.31−6.37)	1.19 (1.18−1.20)
Eastern Asia	901.84 (900.18−903.94)	174.01 (173.70−174.39)	46.10 (46.04−46.20)	33.76 (33.69−33.85)	28.84 (28.79−28.91)	5646.68 (5635.38−5659.81)	63.26 (63.19−63.33)	8.58 (8.56−8.60)	1.64 (1.64−1.65)
Eastern Africa	55.76 (55.23−56.22)	118.25 (117.32−119.25)	59.04 (58.72−59.31)	7.20 (7.14−7.26)	2.08 (2.06−2.09)	4052.48 (4014.11−4086.74)	56.46 (56.10−56.83)	5.70 (5.65−5.75)	0.60 (0.60−0.61)
Middle Africa	19.47 (19.23−19.74)	105.75 (104.34−107.25)	58.92 (58.45−59.56)	6.08 (6.00−6.16)	0.72 (0.71−0.73)	3598.91 (3550.99−3646.29)	58.28 (57.52−58.96)	5.14 (5.07−5.22)	0.48 (0.48−0.49)
Northern Africa	65.40 (64.90−65.76)	151.32 (150.16−152.23)	59.27 (58.94−59.54)	16.29 (16.15−16.40)	2.24 (2.23−2.26)	5079.26 (5042.47−5109.94)	68.01 (67.73−68.36)	7.27 (7.21−7.31)	1.05 (1.04−1.05)
Southern Africa	19.20 (18.96−19.47)	177.86 (175.61−180.60)	58.46 (57.97−59.10)	7.42 (7.32−7.53)	0.66 (0.65−0.67)	5844.49 (5771.08−5928.85)	69.94 (69.34−70.53)	8.67 (8.57−8.79)	0.67 (0.66−0.68)
Western Africa	44.00 (43.55−44.34)	99.39 (98.32−100.22)	60.62 (60.26−60.95)	6.03 (5.97−6.08)	1.60 (1.59−1.61)	3360.99 (3326.51−3385.32)	60.21 (59.72−60.56)	4.87 (4.82−4.91)	0.46 (0.45−0.46)
Caribbean	14.35 (14.10−14.58)	147.23 (144.68−149.68)	40.77 (40.17−41.22)	17.01 (16.65−17.28)	0.47 (0.46−0.47)	4788.77 (4704.73−4866.22)	58.16 (57.51−58.66)	7.32 (7.17−7.42)	1.10 (1.08−1.12)
Central America	29.32 (29.00−29.63)	87.26 (86.33−88.27)	45.65 (45.30−46.00)	9.66 (9.54−9.78)	1.00 (0.99−1.01)	2905.49 (2873.21−2937.37)	56.83 (56.48−57.26)	4.34 (4.29−4.40)	0.67 (0.66−0.68)
South-Eastern Asia	246.01 (245.02−246.97)	166.60 (165.90−167.25)	63.83 (63.64−63.98)	16.53 (16.47−16.61)	8.43 (8.39−8.46)	5619.16 (5597.28−5643.53)	73.53 (73.39−73.71)	7.96 (7.92−7.99)	1.14 (1.14−1.15)
South-Central Asia	474.79 (473.35−475.96)	119.11 (118.72−119.39)	67.56 (67.43−67.67)	10.16 (10.13−10.18)	16.65 (16.60−16.69)	4076.68 (4064.51−4086.89)	73.92 (73.78−74.02)	5.67 (5.66−5.69)	0.75 (0.74−0.75)
Western Asia	84.08 (83.57−84.57)	161.82 (160.87−162.78)	56.65 (56.41−56.92)	22.15 (22.00−22.35)	2.80 (2.78−2.82)	5152.76 (5119.74−5181.90)	67.23 (66.88−67.53)	8.13 (8.08−8.18)	1.25 (1.24−1.26)
Eastern Europe	213.67 (212.72−214.74)	248.16 (247.12−249.39)	56.15 (56.01−56.31)	17.16 (17.08−17.24)	6.69 (6.66−6.72)	7954.95 (7921.32−7996.25)	70.43 (70.31−70.57)	12.06 (12.01−12.12)	1.36 (1.35− 1.36)
Northern Europe	42.47 (42.04−42.88)	131.87 (130.46−133.08)	28.43 (28.17−28.68)	35.21 (34.75−35.53)	1.30 (1.29−1.32)	4154.30 (4103.55−4194.87)	45.96 (45.59−46.27)	6.74 (6.68−6.81)	1.42 (1.40−1.43)
Southern Europe	82.78 (82.26−83.43)	160.97 (159.95−162.18)	32.95 (32.81−33.14)	41.19 (40.86−41.48)	2.57 (2.55−2.59)	5111.96 (5071.54−5150.04)	51.56 (51.34−51.79)	8.09 (8.04−8.15)	1.70 (1.69−1.72)
Western Europe	103.80 (103.12−104.42)	158.74 (157.70−159.72)	33.34 (33.20−33.49)	38.53 (38.17−38.83)	3.21 (3.19−3.23)	5060.63 (5027.97−5094.64)	51.77 (51.58−51.94)	7.95 (7.90−8.00)	1.69 (1.68−1.70)
Australia & New Zealand	10.61 (10.40−10.80)	120.74 (118.04−122.87)	29.93 (29.37−30.28)	39.50 (38.54−40.34)	0.33 (0.32−0.34)	3852.93 (3750.83−3918.11)	48.18 (47.48−48.64)	6.13 (6.02−6.24)	1.41 (1.37−1.43)
Melanesia	2.62 (2.54−2.71)	154.04 (148.06−158.83)	63.78 (62.27−65.30)	11.56 (11.14−11.99)	0.09 (0.09−0.09)	5136.62 (4958.02−5311.80)	68.33 (66.29−70.19)	7.45 (7.16−7.69)	0.87 (0.84− 0.90)
South America	129.99 (129.23−130.61)	133.72 (132.95−134.34)	45.94 (45.75−46.14)	15.05 (14.96−15.14)	4.26 (4.24−4.28)	4351.11 (4325.31−4374.82)	61.14 (60.95−61.38)	6.66 (6.63−6.70)	0.98 (0.98−0.99)
Micronesia & Polynesia	0.32 (0.29−0.35)	204.90 (189.96−228.69)	56.25 (52.85−60.41)	37.93 (34.48−42.89)	0.01 (0.01−0.01)	6434.28 (5907.39−7260.72)	70.66 (67.26−73.35)	10.22 (9.41−11.26)	1.97 (1.81−2.20)
Women									
World	2163.64 (2161.21−2166.91)	119.00 (118.85−119.19)	50.23 (50.19−50.27)	22.18 (22.15−22.22)	75.49 (75.39−75.61)	4182.80 (4176.78−4189.46)	67.08 (67.02−67.12)	5.57 (5.56−5.58)	1.16 (1.16−1.17)
Very high HDI	582.76 (581.42−584.48)	115.25 (114.97−115.59)	35.64 (35.58−35.71)	36.26 (36.16−36.34)	19.00 (18.95−19.05)	3919.22 (3908.65−3929.10)	55.96 (55.88−56.03)	5.55 (5.54−5.57)	1.39 (1.39−1.39)
High HDI	826.19 (824.61−827.56)	109.67 (109.45−109.86)	51.47 (51.40−51.54)	26.95 (26.89−26.99)	28.44 (28.38−28.50)	3823.49 (3815.55−3832.65)	67.94 (67.87−68.01)	5.18 (5.17−5.19)	1.16 (1.15−1.16)
Medium HDI	534.08 (532.64−535.54)	124.16 (123.84−124.51)	70.14 (70.05−70.25)	15.06 (15.02−15.10)	19.53 (19.49−19.59)	4440.94 (4429.97−4454.63)	77.88 (77.78−77.98)	5.69 (5.67−5.71)	0.97 (0.97−0.98)
Low HDI	220.61 (220.03−221.48)	149.14 (148.74−149.65)	72.10 (71.97−72.28)	14.37 (14.32−14.42)	8.52 (8.50−8.56)	5450.95 (5434.63−5471.70)	73.10 (72.96−73.32)	6.68 (6.66−6.70)	1.00 (1.00−1.00)
Northern America	126.78 (126.12−127.46)	110.70 (110.09−111.26)	38.09 (37.89−38.25)	29.84 (29.67−30.01)	4.07 (4.04−4.09)	3728.94 (3705.80−3749.30)	57.56 (57.35−57.75)	5.38 (5.35−5.40)	1.32 (1.31−1.32)
Eastern Asia	490.33 (489.02−491.45)	95.58 (95.30−95.80)	41.22 (41.12−41.31)	37.30 (37.19−37.43)	16.29 (16.24−16.33)	3260.73 (3249.89−3270.14)	60.88 (60.79−60.96)	4.61 (4.60−4.62)	1.16 (1.15−1.16)
Eastern Africa	101.46 (100.86−102.08)	183.43 (182.19−184.52)	71.33 (71.15−71.51)	17.48 (17.35−17.61)	3.95 (3.93−3.97)	6700.22 (6660.74−6744.77)	71.73 (71.51−71.95)	8.16 (8.10−8.21)	1.30 (1.30−1.31)
Middle Africa	28.49 (28.14−28.80)	138.08 (136.41−139.49)	70.74 (70.34−71.18)	11.27 (11.12−11.39)	1.10 (1.08−1.11)	4995.94 (4935.71−5051.69)	70.07 (69.56−70.63)	6.26 (6.19−6.33)	0.84 (0.83−0.85)
Northern Africa	60.34 (59.83−60.77)	130.66 (129.52−131.60)	64.15 (63.84−64.43)	23.39 (23.23−23.58)	2.23 (2.21−2.24)	4690.65 (4647.99−4728.35)	72.88 (72.54−73.23)	5.99 (5.93−6.03)	1.18 (1.17−1.19)
Southern Africa	22.84 (22.55−23.11)	170.14 (167.67−172.01)	61.67 (61.23−62.15)	11.39 (11.26−11.54)	0.86 (0.85−0.87)	6200.10 (6117.88−6272.30)	75.04 (74.60−75.41)	7.64 (7.53−7.72)	1.05 (1.03−1.06)
Western Africa	73.68 (73.24−74.24)	147.62 (146.70−148.72)	74.35 (74.12−74.71)	10.92 (10.85−11.01)	2.84 (2.82−2.86)	5364.61 (5332.64−5405.84)	75.86 (75.56−76.23)	6.65 (6.60−6.70)	0.82 (0.82−0.83)
Caribbean	14.15 (13.94−14.39)	134.23 (132.23−136.58)	48.34 (47.67−48.95)	25.98 (25.56−26.35)	0.49 (0.49−0.50)	4740.95 (4670.23−4827.68)	65.54 (64.89−66.15)	6.25 (6.16−6.35)	1.36 (1.34−1.38)
Central America	39.85 (39.44−40.25)	101.61 (100.56−102.70)	57.32 (56.95−57.63)	20.83 (20.60−21.05)	1.43 (1.42−1.45)	3591.07 (3558.70−3632.79)	69.63 (69.17−70.02)	4.76 (4.71−4.81)	1.04 (1.03− 1.05)
South-Eastern Asia	213.59 (212.56−214.39)	134.70 (134.08−135.20)	64.59 (64.44−64.72)	22.49 (22.36−22.59)	7.64 (7.61−7.68)	4795.35 (4773.37−4815.67)	75.56 (75.40−75.73)	6.21 (6.17−6.23)	1.25 (1.24−1.25)
South-Central Asia	462.05 (460.74−463.70)	116.52 (116.19−116.92)	71.73 (71.60−71.82)	14.59 (14.54−14.64)	16.90 (16.85−16.96)	4172.07 (4160.21−4187.07)	78.86 (78.76−78.97)	5.34 (5.32−5.35)	0.92 (0.92−0.92)
Western Asia	60.14 (59.59−60.69)	116.08 (115.00−117.20)	57.25 (56.94−57.65)	29.79 (29.46−30.11)	2.17 (2.15−2.19)	4069.55 (4040.81−4107.79)	69.66 (69.38−69.97)	5.45 (5.40−5.50)	1.21 (1.20−1.23)
Eastern Europe	149.56 (148.58−150.49)	143.49 (142.71−144.35)	47.21 (47.02−47.41)	25.74 (25.57−25.86)	4.86 (4.83−4.89)	4938.90 (4915.83−4974.47)	65.14 (64.95−65.34)	6.78 (6.74−6.82)	1.36 (1.35−1.37)
Northern Europe	37.33 (36.92−37.70)	113.84 (112.54−114.96)	28.40 (28.11−28.65)	48.92 (48.32−49.44)	1.19 (1.17−1.20)	3752.00 (3708.25−3802.09)	47.32 (46.96−47.64)	5.65 (5.59− 5.70)	1.43 (1.41−1.45)
Southern Europe	57.95 (57.45−58.41)	109.31 (108.29−110.11)	30.15 (29.93−30.38)	54.16 (53.56−54.75)	1.87 (1.86−1.89)	3682.18 (3646.67−3718.96)	50.76 (50.49−50.98)	5.31 (5.26− 5.35)	1.42 (1.41− 1.43)
Western Europe	77.59 (77.10−78.16)	117.44 (116.56−118.40)	30.53 (30.37−30.73)	51.72 (51.26−52.29)	2.47 (2.45−2.49)	3920.31 (3889.39−3954.38)	50.56 (50.38−50.81)	5.74 (5.70−5.78)	1.53 (1.51−1.54)
Australia & New Zealand	9.02 (8.84−9.20)	100.91 (98.99−102.92)	32.51 (32.05−33.06)	52.42 (51.28−54.04)	0.29 (0.29−0.30)	3388.23 (3321.43−3457.00)	52.75 (52.09−53.39)	4.94 (4.85−5.03)	1.35 (1.33−1.38)
Melanesia	3.64 (3.54−3.73)	197.79 (192.44−203.35)	73.69 (72.63−75.16)	25.54 (24.90−26.32)	0.14 (0.13−0.14)	7145.43 (6984.20−7338.76)	78.50 (77.21−79.89)	8.86 (8.60−9.12)	1.61 (1.56−1.65)
South America	134.61 (133.93−135.31)	125.25 (124.62−125.89)	50.57 (50.38−50.76)	24.97 (24.83−25.12)	4.71 (4.68−4.73)	4394.28 (4369.29−4416.01)	67.51 (67.34−67.66)	5.87 (5.85−5.90)	1.26 (1.26−1.27)
Micronesia & Polynesia	0.24 (0.20−0.26)	158.81 (137.05−173.43)	59.34 (55.36−64.21)	53.70 (45.46−61.47)	0.01 (0.01−0.01)	5346.21 (4704.73−5868.01)	75.99 (72.77−79.26)	7.53 (6.48−8.41)	1.97 (1.73−2.17)

^^^Probability in all deaths referred to the proportion of cancer-related deaths relative to all-cause mortality. ^*^Probability in all ages referred to the proportion of premature cancer deaths relative to total cancer deaths. *ASR* age-standardized rate, *HDI* human development index, *PGLEs* potential gains in life expectancy, *UI* uncertainty interval, *YLLs* years of life lost

### Regional variations in distribution patterns

On a global scale, the ASR for cancer-related premature deaths was higher in regions with very high [134.71/10^5^ (95% UI 134.46–134.91)] and high [138.69/10^5^ (95% UI 138.51–138.83)] HDI levels compared to regions with medium [124.64/10^5^ (95% UI 124.43–124.84)] and low [128.59/10^5^ (95% UI 128.15–128.94)] HDI levels. However, premature deaths as a proportion of total cancer deaths were higher in regions with medium [68.47% (95% UI 68.39–68.54)] and low [67.06% (95% UI 66.97–67.18)] HDI levels compared to regions with very high [37.01% (95% UI 36.95–37.04)] and high [51.87% (95% UI 51.82–51.92)] HDI levels. The direction of correlations between the HDI and cancer-related premature death indicators also varied between the sexes. For men, both the probability of cancer-related premature deaths and ASR of YLLs caused by cancer-related premature deaths were higher in regions with very high and high HDI levels compared to regions with medium and low HDI levels. Women showed the opposite patterns. The PGLEs increased with higher HDI levels in men (*P* < 0.05), but the trend was not statistically significant in women (Table 1).

**Fig. 1:**
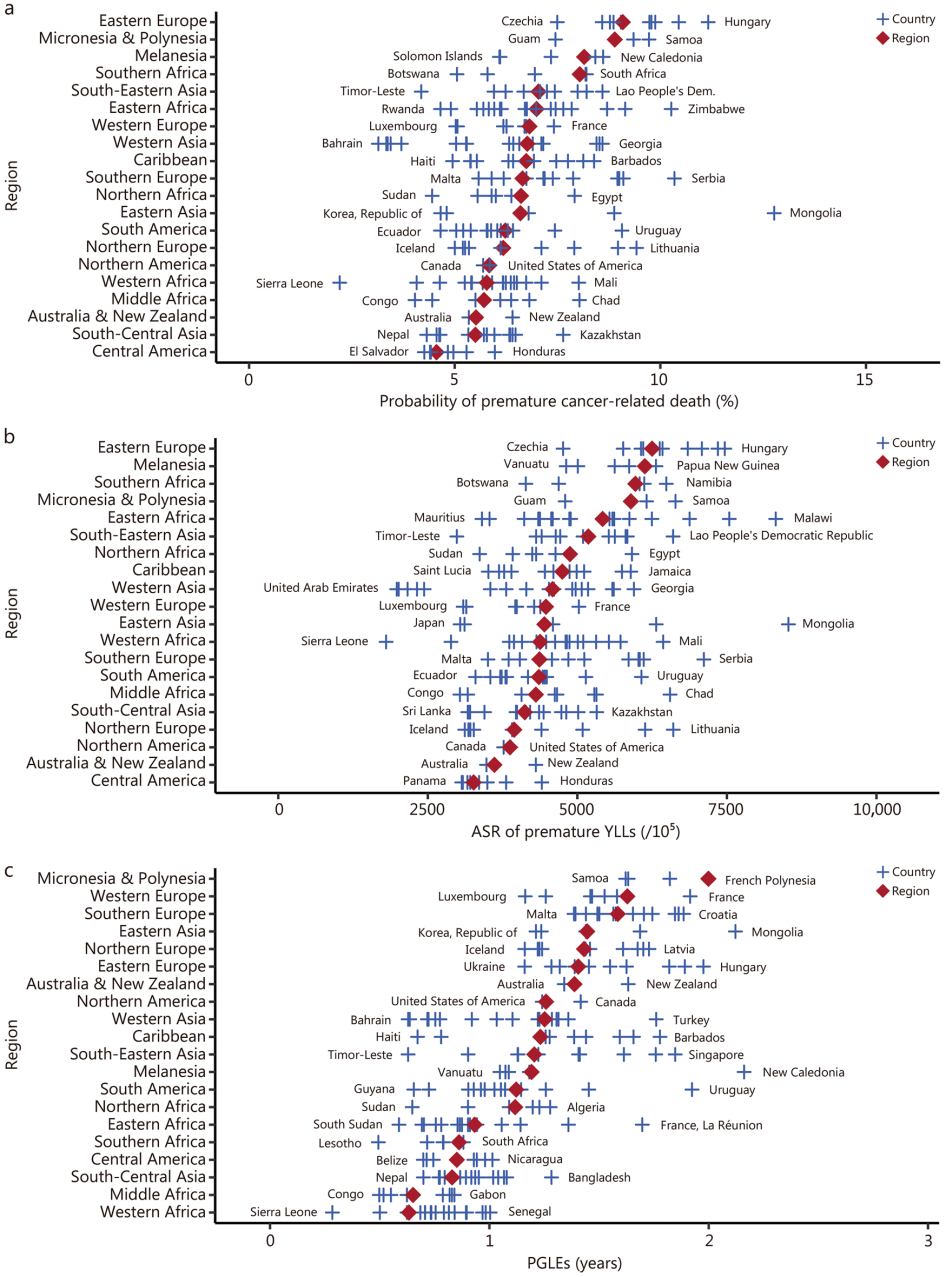
The landscape of premature cancer death indicators in 2022 by region and country, both sexes. **a** Probability of premature cancer death. **b** The ASR of YLLs caused by cancer-related premature death. **c** The PGLEs resulting from the elimination of premature cancer death. Diamonds indicate the average of the indicator in each selected geographic region. Plus signs represent the estimated indicator in each selected country, with the name of the country representing the one with the lowest (left) or the highest (right) value within that geographic region. ASR age-standardized rate, PGLEs potential gains in life expectancy, YLLs years of life lost

On a regional scale, an approximately 2-fold variation was observed in the ASRs for cancer-related premature deaths across sub-regions. Eastern Europe had the highest ASR for cancer-related premature deaths [188.76/10^5^ (95% UI 188.15–189.41)], and Central America had the lowest [94.80/10^5^ (95% UI 94.13–95.60)]. The variations in both the probability of cancer-related premature deaths and ASR of YLLs caused by cancer-related premature deaths across sub-regions followed a similar pattern. Eastern Europe had the highest values [9.09% (95% UI 9.06–9.12) and 6248.92/10^5^ (95% UI 6227.22–6270.13)], and Central America had the lowest [4.56% (95% UI 4.53–4.60) and 3266.31/10^5^ (95% UI 3235.37−3297.19)]. There was a 3.17-fold variation in PGLEs across sub-regions. Micronesia & Polynesia had the highest PGLEs [2.00 years (95% UI 1.80–2.17)], and Western Africa had the lowest [0.63 years (95% UI 0.63–0.64)] (Table 1; Fig. 1).

On a national scale, there were more pronounced disparities. The ASRs for cancer-related premature deaths ranged from 48.31/10^5^ (95% UI 45.32−51.57) in Sierra Leone to 264.54/10^5^ (95% UI 255.22−274.73) in Mongolia. Cancer-related premature deaths as a proportion of total cancer deaths varied from 18.31% (95% UI 18.20–18.43) in Japan to 84.44% (95% UI 76.10–91.16) in São Tomé and Príncipe (Additional file 2: Table S2). The distributions of the ASRs of YLLs and PGLEs due to cancer-related premature deaths followed a comparable pattern. Sierra Leone had the lowest ASR of YLLs [1803.20/10^5^ (95% UI 1693.44−1917.40)] and PGLEs [0.28 years (95% UI 0.27–0.30)], Mongolia had the highest ASR of YLLs [8528.72/10^5^ (95% UI 8217.52−8837.04)], and New Caledonia registered the highest PGLEs [2.16 years (95% UI 1.87–2.46)] (Additional file 2: Table S3; Fig. 1).

### The distribution patterns by cancer site

**Fig. 2:**
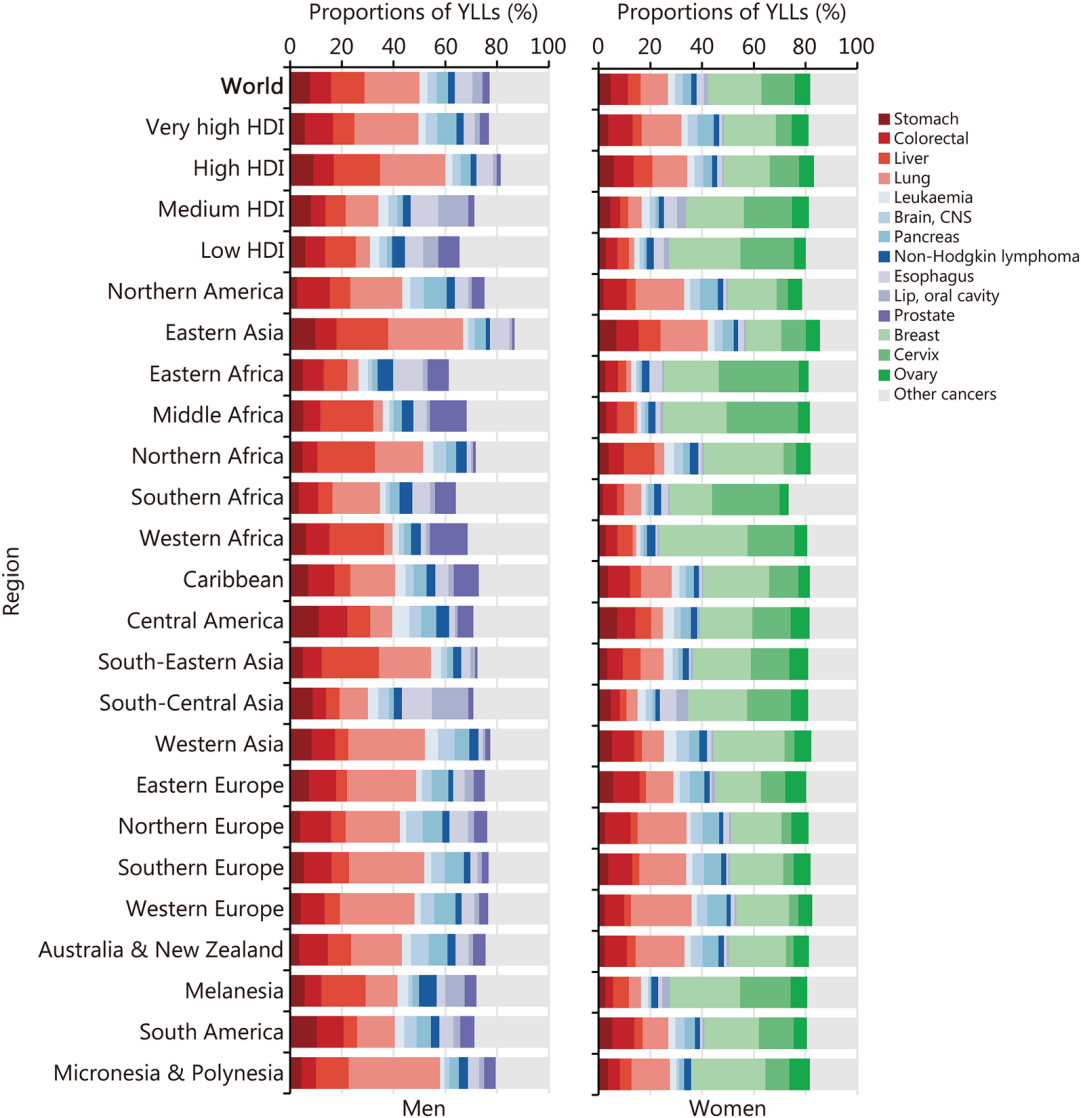
The proportions of years of life lost (YLLs) attributable to cancer-related premature deaths in 2022, by cancer site, region, and sex. CNS central nervous system, HDI human development index

On a global scale, the burden of premature deaths resulting from specific cancer sites differed by sex. Lung cancer emerged as the leading cause of cancer-related premature deaths in men (YLL = 18,854.77 thousand, 95% UI 18,806.30–18,902.94), contributing approximately 21.34% (95% UI 21.28–21.40) of the total YLLs. Liver cancer [12.79% (95% UI 12.75–12.84)] and colorectal cancer [8.23% (95% UI 8.19–8.27)] ranked second and third. For women, breast cancer was the leading cause of cancer-related premature deaths (YLL = 15,581.79 thousand, 95% UI 15,536.09–15,633.70), accounting for 20.64% (95% UI 20.57–20.71) of the total YLLs, followed by cervical cancer [12.95% (95% UI 12.89–13.00)] and lung cancer [10.48% (95% UI 10.44–10.52)] (Fig. 2; Additional file 2: Tables S4 and S5).

The predominant cancer sites varied across regions with different HDI levels. Among men, lung [24.75% (95% UI 24.63–24.89)], colorectal [10.96% (95% UI 10.88–11.04)], liver [8.22% (95% UI 8.15–8.29)] and pancreatic [7.41% (95% UI 7.35–7.47)] cancers were the leading causes of cancer-related premature deaths in very high HDI regions. In contrast, liver [11.82% (95% UI 11.63–12.02)], prostate [8.29% (95% UI 8.15–8.42)], colorectal [7.67% (95% UI 7.51–7.82)], and esophageal [7.12% (95% UI 6.98–7.27)] cancers were the leading causes in low HDI regions (Fig. 2; Additional file 2: Table S5).

Among women, breast [20.05% (95% UI 19.93–20.18)], lung [15.30% (95% UI 15.18–15.41)], colorectal [9.34% (95% UI 9.26–9.43)], and ovarian [6.54% (95% UI 6.47–6.61)] cancers were the leading causes of cancer-related premature deaths in regions with a very high HDI level. Cervical cancer [6.10% (95% UI 6.03–6.18)] was only fifth in these regions. In high HDI regions, cervical cancer [11.27% (95% UI 11.19–11.35)] climbed to the third position, following breast [17.85% (95% UI 17.74–17.95)] and lung [13.43% (95% UI 13.35–13.51)] cancers. In medium HDI regions, breast [22.21% (95% UI 22.06–22.35)] and cervical [18.67% (95% UI 18.54–18.81)] cancer ranked first and second, and collectively accounted for over 40% of the total YLLs due to cancer-related premature deaths. In low HDI regions, this proportion increased to 48% (Fig. 2; Additional file 2: Table S5).

We reported the leading cause of YLLs due to cancer-related premature deaths, categorized by cancer site, for 185 countries. Among men, lung cancer ranked first in 97 countries (52.43%), liver cancer in 36 countries (19.46%), stomach cancer in 18 countries (9.73%), colorectal cancer in 11 countries (5.95%), esophageal cancer in 7 countries (3.78%) and prostate cancer in 6 countries (3.24%). Kaposi’s sarcoma (KS) ranked first in 8 African countries (Botswana, Eswatini, Lesotho, Mozambique, Namibia, Uganda, Zambia, and Zimbabwe). Oral cancer ranked first in India and Pakistan. For women, breast cancer ranked first in 121 countries (65.41%), cervical cancer in 41 countries (22.16%), and lung cancer in 21 countries (11.35%). Esophageal cancer was the primary cause in Bangladesh, and liver cancer ranked first in Mongolia (Additional file 2: Table S6).

**Fig. 3:**
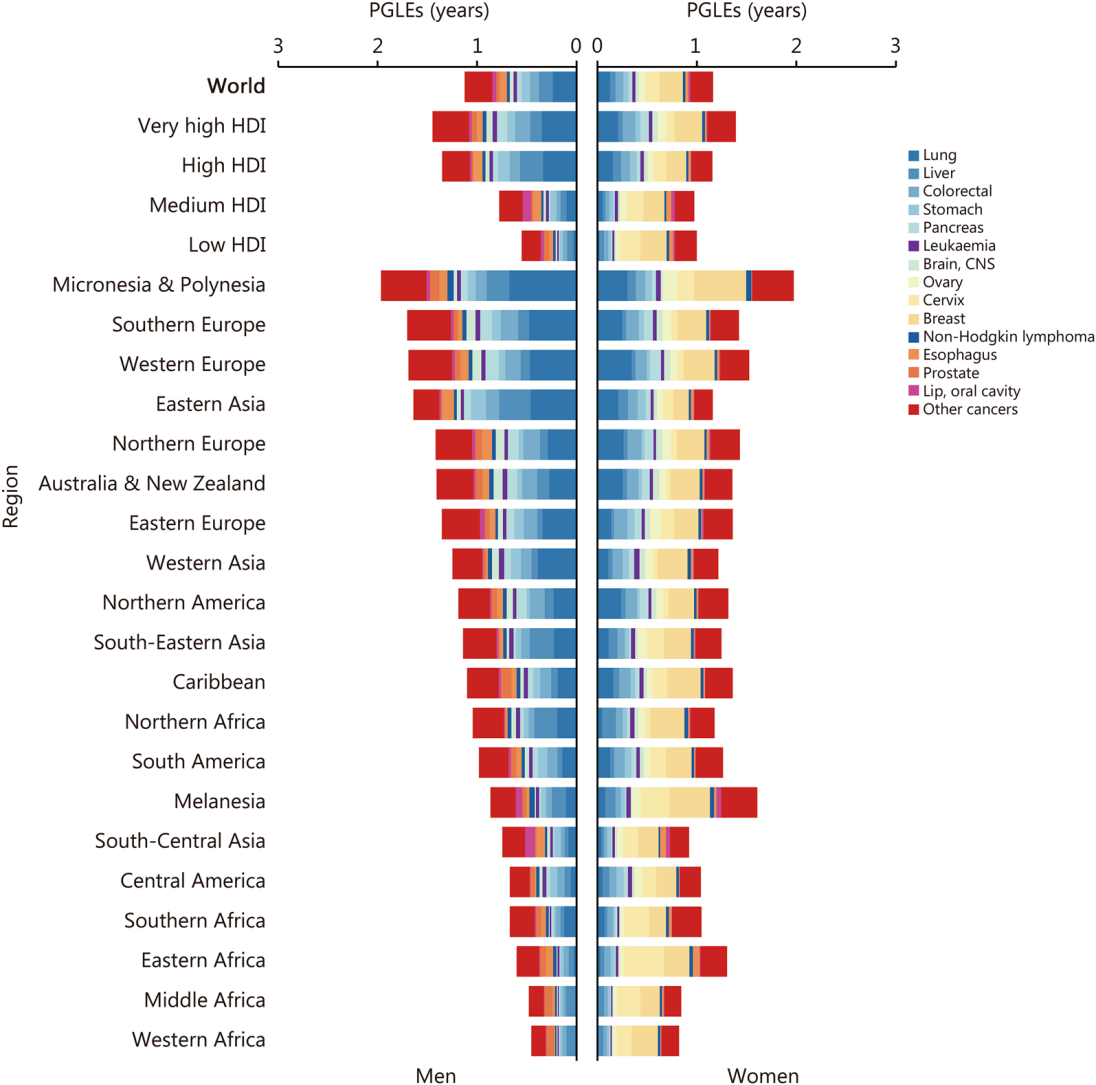
The potential gains in life expectancy (PGLEs) by eliminating premature cancer deaths in 2022, by cancer site, region, and sex. CNS central nervous system, HDI human development index

We estimated the PGLEs categorized by cancer sites. For men, we found that by eradicating the 4 leading causes (lung, liver, colorectal, and stomach cancer) of cancer-related premature deaths, we could achieve 0.55 years (95% UI 0.55–0.55) of PGLEs, accounting for 48.67% of the total PGLEs that would be gained by eliminating all cancer-related premature deaths. The most significant gain was observed in Micronesia & Polynesia, where the PGLEs would increase by 1.10 years (95% UI 0.82–1.41), followed by Eastern Asia [1.07 years (95% UI 1.05–1.08)], Southern Europe [0.85 years (95% UI 0.84–0.86)], and Western Europe [0.78 years (95% UI 0.76–0.80)]. For women, eradicating the 3 leading causes (breast, cervical, and lung cancers) of cancer-related premature deaths could give 0.49 years (95% UI 0.49–0.49) of PGLEs, accounting for 42.24% of the total PGLEs. Eradicating premature deaths caused by breast and cervical cancers alone could substantially increase life expectancy in Melanesia [0.69 years (95% UI 0.65–0.73)], Micronesia & Polynesia [0.69 years (95% UI 0.49–0.94)], and Eastern Africa [0.65 years (95% UI 0.64–0.65)] (Fig. 3; Additional file 2: Table S7). The country-level analysis showed that men in Mongolia would gain the greatest increase in life expectancy from elimination of liver cancer-related premature deaths [0.96 years (95% UI 0.89–1.02)], and women in Malawi would experience the most significant improvement by eradicating cervical cancer-related premature deaths [0.87 years (95% UI 0.84–0.90)] (Additional file 2: Table S8).

### Twenty-year trends at the country level

**Fig. 4:**
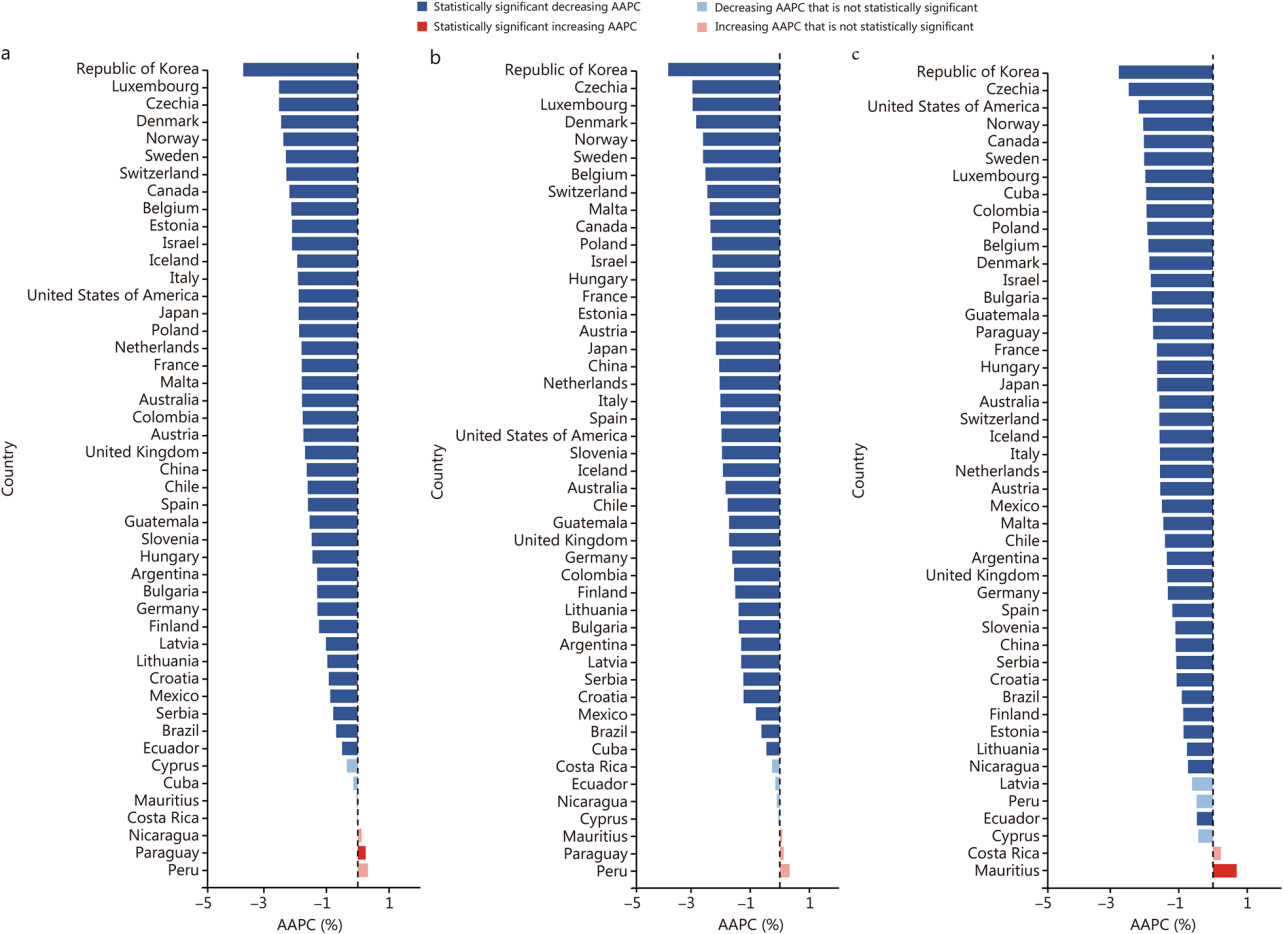
The average annual percent changes (AAPCs) for premature cancer death indicators from 2003 to 2022 by country, both sexes. **a** Probability of premature cancer-related death. **b** The ASR of YLLs caused by cancer-related premature death. **c** The PGLEs resulting from the elimination of premature cancer death. ASR age-standardized rate, PGLEs potential gains in life expectancy, YLLs years of life lost

There were 47 countries with 20 years of cancer-related premature mortality data. The indicators for cancer-related premature deaths all decreased significantly in 38 countries (*P* for AAPCs < 0.05). The Republic of Korea showed the fastest decline in the probability of premature cancer deaths [-3.66% (95% CI - 3.72 to - 3.60)], the ASR of YLLs [- 3.77% (95% CI - 3.84 to - 3.71)], and the PGLEs [- 2.75% (95% CI - 3.17 to - 2.32)] (Fig. 4; Additional file 1: Table S5). The probability of premature cancer deaths [0.58% (95% CI 0.25−0.92)] and the ASR of YLLs [0.38% (95% CI 0.09−0.67)] increased significantly only among men in Paraguay. The PGLEs increased significantly among women in both Costa Rica [0.42% (95% CI 0.08−0.76)] and Mauritius [0.78% (95% CI 0.41−1.15)] (Additional file 1: Tables S6-S7 and Figs. S1-S2).

The probability of premature cancer death in 2022 compared with 2015 decreased by more than 15.50% in 16 countries: specifically, Canada (15.67%), France (15.75%), Norway (15.96%), Czech Republic (16.46%), China (16.77%), Poland (17.18%), Netherlands (17.69%), Israel (17.70%), Sweden (17.75%), Switzerland (19.59%), Luxembourg (19.73%), Denmark (19.74%), Estonia (20.27%), Malta (21.93%), Republic of Korea (22.54%) and Belgium (22.66%). When analyzed by sex, 26 countries achieved a reduction of more than 15.50% premature cancer death probabilities among men, but only 12 among women (Additional file 1: Table S5 and Figs. S3-S5).

## Discussion

This was a comprehensive study examining the probability of premature death, YLLs, and PGLEs due to premature cancer deaths by world region, HDI level, sex, and cancer site. We found the probability of premature cancer deaths was 6.49%. Overall, 163.86 million years were lost to premature cancer deaths, and the PGLEs were 1.16 years worldwide. In high HDI regions, lung and colorectal cancer were the top causes of premature death for men, with breast and lung cancer for women. In low HDI regions, liver cancer was the top cause of premature deaths among men, and breast and cervical cancer were the main types that led to premature deaths among women. Most countries with high-quality consecutive mortality data showed declining trends in cancer-related premature deaths, with 16 countries achieving substantial reductions from 2015 to 2022. Using these indicators, we clarified the priorities for the prevention and control of different cancers in specific regions based on the public health impact of premature deaths.

Discussions about the definition of premature death have focused on age limitations. One approach defines premature death as occurring before the age of 70 years [1, 19]. Another considers it as death occurring before the approximate average age of death in a given country over the study period, which, for instance, is 75 years in some developed countries [20]. In this study, we considered premature deaths on a global scale. The world’s average life expectancy was 71.79 years in 2022, and the incidence of cancer before the age of 30 years is exceptionally low because of the biological nature of oncogenesis. More significantly, the WHO’s SDG Target 3.4 sets the age bracket for premature deaths at 30 to 69 years. To facilitate more accurate global comparisons, we therefore chose to define premature death as occurring within this same age range of 30−69 years.

The disparity in the disease spectrum and premature mortality among populations with varying socioeconomic statuses has been extensively debated. The Global Burden of Disease data indicate that communicable, maternal, neonatal, and nutritional diseases constitute a substantial proportion of YLLs in many low-income countries. However, in high-income countries, NCDs, including cancer, are the foremost public health challenge[21]. A national cohort study in the USA reported a dose-response relationship between the cumulative number of unfavorable social determinants of health and elevated rates of premature all-cause mortality[20]. However, the distribution of cancer-related mortality burdens may have distinct patterns and may therefore warrant a dedicated and separate investigation.

In this current study, we calculated 6 indicators to provide a comprehensive portrayal of cancer-related premature death: 1) the number of deaths, representing the estimated actual count of fatalities; 2) the ASR of mortality, adjusting for population age structure; 3) the number of YLLs, which quantifies the life years lost due to cancer-related premature death within a specific population; 4) the ASR of YLLs, which standardizes the YLLs rate to account for differences in age distribution; 5) the probability of death; and 6) the PGLE upon eliminating targeted deaths, which is considered as a clear indicator of the impact of targeted deaths on public health [22]. Regions with very high and high HDI levels showed higher numbers of premature cancer deaths, elevated age-standardized mortality rates of cancer, and a greater proportion of premature deaths attributable to cancer compared with regions with relatively low HDI levels. This aligns with global observations derived from cancer surveillance data across the entire population [7]. However, premature cancer deaths as a proportion of all cancer deaths were higher in low HDI than in high HDI regions. This suggests that although the total number of cancer deaths in these regions was low, a larger proportion of cancer deaths occurred at younger ages. We found comparable trends in YLLs across regions. We also found that PGLEs had a positive relationship with HDI levels. Regions with high concentrations of transitioned countries, such as Europe, Eastern Asia, and Australia & New Zealand, had higher PGLEs than other regions. This indicates that cancer-related premature deaths have a more pronounced impact on populations living in relatively rich countries. This is because individuals in these nations are more likely to die of cancer than other diseases. This discrepancy can be attributed to factors such as local disease detection, diagnosis capabilities, and competitive death risks, all of which are influenced by the local socioeconomic level. However, before undertaking any data comparisons across regions, it is crucial that we understand the influence exerted by socioeconomic status on the quality of surveillance data. Specifically, transitioning countries may allocate fewer resources to disease surveillance, compromising the quality of the data they generate. In contrast, data reported by transitioned countries are more likely to accurately mirror the true state of affairs. This discrepancy has the potential to distort the inherent correlation between the HDI and disease burden, potentially giving rise to incorrect interpretations.

After stratifying by sex, we found that PGLEs were higher in women than men, both globally and in almost all regions with low HDI regions, including Africa, the Caribbean, Central America, South-Eastern Asia, and South-Central Asia. The opposite was true in high HDI regions, including Southern Europe, Western Europe, and Australia & New Zealand, although there were a few exceptions. These findings suggest that in resource-limited regions, the potential benefits of preventing premature cancer-related deaths may be greater for women. Upon initial inspection, this result appeared somewhat perplexing, given that all previous studies have consistently indicated a higher incidence and greater mortality rate among men than women [7, 23]. However, the variations in YLLs and PGLEs across regions and between sexes mirror the differences in the cancer spectrum, which in turn reflects the varying prevalence of cancer-related risk factors across different populations. For instance, infection-related cancers are mostly seen in low HDI regions, whereas cancers associated with reproductive, dietary, and hormonal factors are mostly seen in high HDI regions [17]. However, liver and cervical cancers, two of the most significant infection-related cancers, tend to manifest at younger ages than other types of cancers [24, 25]. We therefore analyzed YLLs and PGLEs by cancer site in each region and found intriguing results.

We found that breast cancer was the leading cause of premature cancer deaths in women, regardless of HDI levels. Globally, breast cancer is the most frequently diagnosed cancer and the primary cause of cancer-related deaths in women by 2022 [7]. In developed countries, breast cancer has shown both an increased incidence rate and a decrease in mortality rates. Conversely, transitioning countries show opposing trends, which can be attributed to a combination of shifting risk factors, improvements in screening efforts, and advancements in diagnostic and treatment technologies [26]. The incidence of breast cancer in women demonstrates distinctive age distribution patterns, characterized by an earlier onset and an inverted bell-shaped age curve that peaks between 45 and 65 years of age, with notable racial disparities [27–29]. This unique pattern distinguishes it from the distribution of other cancers, in which the incidence rates generally increase with age. Breast cancer incidence rates among younger females are highest in regions with very high HDI levels, whereas mortality rates peak in low and medium HDI regions [30]. In recent years, studies have also observed steeper increases in breast cancer incidence among young women across diverse populations [31–34], and younger patients tend to have higher breast cancer mortality [35]. All of this has resulted in breast cancer becoming the leading cause of cancer-related premature death in nearly all regions across the globe.

Cervical cancer is one of the most significant threats to women in low HDI regions, particularly in Africa. The total YLLs due to cervical and breast cancers are more than half the overall YLLs in Eastern, Middle, and Western Africa in women. Our results showed that preventing premature deaths from cervical cancer alone could result in an additional 0.2 to 0.4 life-years in Africa, accounting for approximately 30% of all cancer-related PGLEs in women in these regions. It indicates that in resource-limited regions, significant public health benefits can therefore be achieved by prioritizing the allocation of resources to control cervical cancer. Fortunately, prevention and control strategies for cervical cancer have been extensively discussed and proven to be effective at affordable costs [36]. The WHO launched the Cervical Cancer Elimination Initiative in 2020 [37], offering a clear pathway for countries to reduce cervical cancer incidence and narrow international disparities associated with this disease. However, the most recent cancer statistics showed great disparities in premature deaths resulting from cervical cancer, suggesting that there is still a long way to go to eliminate this disease globally.

Lung cancer is one of the leading causes of cancer-related premature death in both men and women in high HDI regions. The disease burden of lung cancer is high in Eastern Asia, especially in China [7]. In recent years, China has witnessed a dramatic surge in the incidence rate of lung cancer, particularly among women, whereas mortality rates have declined for both sexes [38]. Premature lung cancer-related deaths are also the highest in women in this region. Potential risk factors for women include passive smoking [39], household air pollution from cooking and heating with solid fuels [40], and outdoor air pollution [41]. There are different risks for men, where smoking is the most prominent risk factor. Lung cancer screening using low-dose computed tomography has been shown to reduce lung cancer-specific mortality in several randomized controlled trials [42]. However, the primary target groups for these programs are heavy smokers, predominantly men [43]. Further research is required to ascertain their efficacy in preventing premature deaths among non-smoking women [44].

Liver cancer ranked first in men in low HDI regions, including Middle Africa, Northern Africa, Western Africa, South-Eastern Asia, and Melanesia. In these regions, hepatitis B virus (HBV) and hepatitis C virus (HCV) continue to be the most important risk factors for liver cancer, with the median age at diagnosis being notably younger than in developed countries [45]. Prevention is key to reducing liver cancer-related mortality, and the effect of HBV vaccination in newborns has already been observed in some countries [46]. However, a recent study found that the prevalence of HBV and HCV infection in patients with cirrhosis in Africa exceeded 50%, suggesting that viral hepatitis was the primary cause of cirrhosis in these regions [47]. Our findings once again underscore the urgent need to implement preventive measures against liver cancer in Africa [48], including vaccination programs, strategies to halt maternal-to-child transmission, the provision of antiretroviral and antiviral treatments, and the reduction of dietary exposure to aflatoxin [49].

The incidence of KS remains relatively low in the general population, but it is disproportionately high among people living with human immunodeficiency virus (HIV) [50]. Previous global analyses have highlighted that KS-related mortality is notably higher in sub-Saharan Africa [51]. Our study further revealed that the majority of these deaths occur prematurely, with children and young adults being the primary victims. This phenomenon was exclusively observed in African countries. The impact of KS on young and middle-aged workers exacerbates the decline in regional prosperity, thereby increasing the vulnerability to diseases across the entire population. On a positive note, this condition has become treatable thanks to the growing availability of antiretroviral therapy and chemotherapy. In low-resource settings, pediatric KS can now be managed to enable long-term survival [52]. To address this significant challenge, active control of the HIV pandemic in affected regions and accelerated efforts to investigate and develop targeted treatments and vaccines against the KS-associated herpesvirus are imperative [53].

To provide a more in-depth understanding of the evolving trend in the burden of cancer-related premature death, we included 47 countries for which consecutive mortality data from 2003 to 2022 were available. The United Nations adopted SDG 3.4 to address the escalating burden of NCDs. This goal called for a reduction of one-third in premature mortality from NCDs by 2030. Cancer is a major NCD, and we therefore applied the same target to evaluate the effectiveness of current cancer prevention and control policies. We found significant declining trends in cancer-related premature deaths in most countries. However, only 16 countries had achieved substantial reductions in the probability of cancer-related premature deaths, suggesting that they might be able to meet the one-third reduction target outlined in SDG 3.4. These results were in line with a previous global study [9]. This study noted that SDG 3.4 was most likely not to be achieved by 2030 in cancers amenable to early detection, such as breast and colorectal cancer. This was in contrast to cancers with well-established primary prevention measures, such as liver and cervical cancer, especially in regions with a low HDI. This observation highlights the complexity and resource-intensive nature of cancer prevention and control compared with other NCDs. However, countries like Japan, which have already achieved substantial reductions in premature cancer mortality in recent years, will have a significant challenge to further decrease the probability of premature death by one-third at the population level. These factors collectively suggest that applying uniform targets to evaluate cancer progress across countries with diverse cancer profiles may not be practical or feasible.

## Strengths and limitations

In this study, we used the latest global cancer statistics to analyze YLL as a metric to quantify the health lost to cancer and PGLE as a metric to quantify the benefits of cancer prevention and control efforts, achieved through the elimination of premature cancer deaths. We also explored the evolving trends in the probability of cancer-related premature death across selected countries. These results will provide useful evidence to inform and support policy-making.

This study had several limitations. First, the accuracy and comparability of estimates for YLLs and PGLEs depend heavily on the quality and quantity of cancer surveillance data and vital statistics available globally. The coverage and quality of cancer registry systems vary across countries, with notable deficiencies in high-quality data in regions such as South America and Africa. GLOBOCAN 2022 and the WHO mortality database are the most up-to-date and available sources of cancer mortality statistics derived from population-based monitoring data, and all the data included in these databases have met rigorous quality control standards [54]. However, it is important to bear in mind that the data contained within these databases are still subject to quality assessment and calibration, and this consideration should be taken into account when interpreting the results. Second, our focus was exclusively on premature cancer-related deaths. We did not consider the premature deaths attributed to other competing risks, and we were therefore unable to assess the impact of these competing risks on cancer mortality. Third, most of the 47 countries with consecutive mortality data have high HDI levels and have sufficient resources to collect cancer and vital surveillance data. The underrepresentation of countries with relatively low HDI levels limits our ability to conduct a global analysis of trends in cancer-related premature death.

## Conclusions

We conducted this study to understand the patterns and trends in cancer-related premature deaths and their effect on population life expectancy on a global scale. We found that the current level of premature deaths attributable to cancer remains substantial and also has pronounced global disparities. Eliminating premature deaths from cancer, particularly from major cancer types, could substantially increase life expectancy. Many countries have seen declining trends in premature cancer mortality in recent years, but achieving the SDG 3.4 goal by 2030 remains challenging. Our study offers scientific evidence to help evaluate current cancer prevention and control policies. Furthermore, it could present recommendations to guide and shape public health policies, enhancing cancer control initiatives and thereby safeguarding individuals during their most productive life stages across a multitude of regions and countries.

## Supplementary Information

**Additional file 1.** Data sources. Methods for calculating indicators. **Table S1** Cancer classification used in analysis. **Table S2** The annual collection status of the mortality data by country, 2003–2022. **Table S3** Data intervals for estimating missing mortality data in specified years, by country. **Table S4** The step-by-step calculation of the life expectancy. **Table S5** AAPCs for premature cancer death indicators by country, 2003–2022. **Table S6** AAPCs for premature cancer death indicators by country among men, 2003–2022. **Table S7** AAPCs for premature cancer death indicators by country among women, 2003–2022. **Table S8** Reduction proportion in probability of premature cancer-related death (2015−2022) by country and sex. **Fig. S1** AAPCs for premature cancer death indicators by country among men, 2003−2022. **Fig. S2** AAPCs for premature cancer death indicators by country among women, 2003−2022. **Fig. S3** The changing trends for the probability of premature cancer-related death from 2003 to 2022 by country and sex. **Fig. S4** The changing trends for the ASR of YLLs caused by cancer-related premature death from 2003 to 2022 by country and sex. **Fig. S5** The changing trends for the PGLEs resulting from the elimination of premature cancer death from 2003 to 2022 by country and sex.

**Additional file 2.** **Table S1** Total cancer deaths, years of life lost (YLLs), and potential gains in life expectancy (PGLEs) by eliminating total cancer-related deaths, by world region, sex, and HDI, 2022. **Table S2** Total cancer deaths and premature cancer deaths, by country and sex, 2022. **Table S3** Years of life lost (YLLs) and potential gains in life expectancy (PGLEs) caused by total cancer deaths and premature cancer deaths, by country and sex, 2022. **Table S4 **Years of life lost (YLLs) and potential gains in life expectancy (PGLEs) caused by total cancer deaths and premature cancer deaths worldwide, by cancer site and sex, 2022. **Table S5** The proportions (%) of years of life lost (YLLs) attributable to cancer-related premature deaths in 2022, by cancer site, region, and sex [% (95% UI)]. **Table S6** The leading cause of years of life lost (YLLs) due to premature cancer deaths, by country and sex, 2022. **Table S7** The potential gains in life expectancy (PGLEs) by eliminating premature cancer deaths in 2022, by cancer site, region, and sex (years, 95% UI). **Table S8** The leading cause of potential gains in life expectancy (PGLEs) attributable to premature cancer deaths, by country and sex, 2022.

## Data Availability

All the data used in this study were obtained from public databases. Cancer data are available online at https://gco.iarc.fr/today/. Population, all-cause mortality, and life expectancy data are available at https://population.un.org/wpp/. Another request for the full dataset is available from the corresponding author.

## Abbreviations

AAPC: Average annual percent change
ASR: Age-standardized rate
CI: Confidence interval
HBV: Hepatitis B virus
HCV: Hepatitis C virus
HDI: Human development index
KS: Kaposi’s sarcoma
HIV: Human immunodeficiency virus
NCD: Noncommunicable disease
PGLE: Potential gain in life expectancy
SDG: Sustainable development goal
UI: Uncertainty interval
WHO: World Health Organization
YLL: Year of life lost
