# Supplementary material for: Global patterns and trends in cancer-related premature death and their impact on life expectancy across 185 countries: a population-based analysis
Source: Mil Med Res. 2025 Sep 3;12:56. doi: 10.1186/s40779-025-00645-9 (PMC12406598; doi:10.1186/s40779-025-00645-9)
Supplement: Supplementary file 1 — Additional file 1. Data sources. Methods for calculating indicators. Table S1 Cancer classification used in analysis. Table S2 The annual collection status of the mortality data by country, 2003–2022. Table S3 Data intervals for estimating missing mortality data in specified years, by country. Table S4 The step-by-step calculation of the life expectancy. Table S5 AAPCs for premature cancer death indicators by country, 2003–2022. Table S6 AAPCs for premature cancer death indicators by country among men, 2003–2022. Table S7 AAPCs for premature cancer death indicators by country among women, 2003–2022. Table S8 Reduction proportion in probability of premature cancer-related death (2015−2022) by country and sex. Fig. S1 AAPCs for premature cancer death indicators by country among men, 2003−2022. Fig. S2 AAPCs for premature cancer death indicators by country among women, 2003−2022. Fig. S3 The changing trends for the probability of premature cancer-related death from 2003 to 2022 by country and sex. Fig. S4 The changing trends for the ASR of YLLs caused by cancer-related premature death from 2003 to 2022 by country and sex. Fig. S5 The changing trends for the PGLEs resulting from the elimination of premature cancer death from 2003 to 2022 by country and sex. [file 40779_2025_645_MOESM1_ESM.pdf]

Data sources

Global cancer mortality data for 2022

All data were obtained from the Global Cancer Observatory (GCO; https://gco.iarc.fr/) established by the International Agency for Research on Cancer (IARC). Cancer mortality data for 39 individual cancer sites and all cancers combined (International statistical classification of diseases and related health problems, 10th revision, code C00 to C97) were obtained from the GLOBOCAN 2022 database for 185 countries, stratified by sex and age. The cancer classification can be found in **Additional file 1: Table S1**. These data were based on the best available cancer and vital statistics data for a given country or region.

Cancer mortality data for 47 countries during 2003 − 2022

We compiled annual mortality data from the World Health Organization (WHO) mortality database (https://www.who.int/data/data-collection-tools/who-mortality-database). For all countries, an analysis of mortality trends was conducted over 20 years, from 2003 to 2022. In the present analysis, only countries were included that had missing mortality data for fewer than 3 years during this period (**Additional file 1: Table S2**).

To acquire high-quality cancer-related mortality data for China, we have extracted cancer mortality data from a series of the China Cancer Registry Annual Reports released by the National Cancer Center of China, which have been documenting cancer surveillance data since the year of 2003. This cancer registry data has frequently been used in prior analyses, aiding in the examination of cancer incidence and mortality trends as well as national estimations.

The calculation of the missing mortality data for each country was based on the trends observed within the most recent 10-year window, employing either extrapolation or interpolation methods [1] (**Additional file 1: Table S3**).

Methods for calculating indicators

YLL

YLL is the most commonly used indicator for quantifying the impact of premature deaths, and is calculated as the loss of life years caused by specific deaths occurring before reaching the expected lifespan [2]. The number of YLL is calculated by summing the number of deaths at each age, or sometimes calculated by summing the number of deaths at each age between 1 − 74 years. We calculated the YLL by summing the number of deaths at each age.

The years of life lost caused by premature deaths were calculated as the YLLs occurring between the ages of 30 and 69.

This calculation is done for deaths due to this cause in each age group, and the results are then summed. Mathematically, these calculations can be summarized as follows [3]:

$$YLL = \sum_{i=0}^{\infty} a_i d_i$$

Here, i represents each age group, usually every 5 years (0 − 4, 5 − 9, …, 80 − 84, ≥ 85). a_i denotes the number of years of life remaining when death occurs, while d_i stands for the number of observed deaths in the population under investigation between ages i and $i+1$.

PGLE

PGLE was computed as the difference between the life expectancy after removing all cancer-related premature deaths and the baseline life expectancy [4, 5].

x represents age.

n stands for the age interval.

P_x refers to the average population.

D_x denotes the number of all-cause deaths.

m_x refers to the mortality rate at age x. The number of deaths at age x divided by the number of person-years at risk at age x.

$$m_x = \frac{D_x}{P_x}$$

q_x refers to the probability of dying at age x. Also known as the (age-specific) risk of death.

$$q_x = \frac{nm_x}{1 + (n - a_x)m_x}$$

a_x refers to the person-years lived in the interval x to $x+n$. Generally, it can be assumed that those dying in the interval are evenly distributed, then the above formula is equivalent to:

$$q_x = \frac{2nm_x}{2 + nm_x}$$

p_x refers to the probability of surviving from exact age x to $x+n$.

$$p_x = 1 - q_x$$

l_x, the survivorship function: the number of people alive at age x. These values are computed recursively from the m_x values using the formula, with l_0, the "radix" of the life table, which is the assumed number of births to age 0 and usually taken as 100,000.

$$l_{x+n} = l_x \times p_x$$

d_x refers to the number of deaths in the interval (x, $x+n$) for persons alive at age x.

$$d_x = l_x - l_{x+n}$$

L_x refers to the total number of person-years lived by the cohort from age x to $x+n$.

$$L_x = L_{x+n} + a_x d_x$$

T(x) refers to the total number of person-years lived by the cohort from age x until all members of the cohort have died.

$$T_x = \sum_{a=x}^{\infty} L_a$$

e_x refers to the (remaining) life expectancy of persons alive at age x.

$$e_x = \frac{T_x}{l_x}$$

The cause-eliminated life expectancy (CELE) is the life expectancy calculated by deducting the influence of certain causes of death according to the above formula.

The PGLEs are the difference between the life expectancy calculated by CELE and the life expectancy calculated by all causes of death.

Probability of premature death

Probability (%) of dying between the ages of 30 and 69. This study was calculated according to the life table of a 5-year-old age group.

$$P = 1 - \prod_{x=30}^{69} (1 - q_x)$$

q_x refers to the probability of dying at age x. P refers to the premature mortality. The steps to calculate life expectancy are shown in **Additional file 1: Table S4**.

Reference

1. Kim HJ, Fay MP, Feuer EJ, Midthune DN. Permutation tests for joinpoint regression with applications to cancer rates. Stat Med. 2000;19(3):335-51.
2. Devleesschauwer B, Havelaar AH, Maertens de Noordhout C, Haagsma JA, Praet N, Dorny P, et al. Calculating disability-adjusted life years to quantify the burden of disease. Int J Public Health. 2014;59(3):565-9.
3. Murray CJ. Quantifying the burden of disease: the technical basis for disability-adjusted life years. Bull World Health Organ. 1994;72(3):429-45.
4. Wright JC, Weinstein MC. Gains in life expectancy from medical interventions-standardizing data on outcomes. N Engl J Med. 1998;339(6):380-6.
5. Arias E, Heron M, Tejada-Vera B. United States life tables eliminating certain causes of death, 1999-2001. Natl Vital Stat Rep. 2013;61(9):1-128.

Table S1 Cancer classification used in analysis

ICD-10 codes	Full title of cancer sites	Short title used in the paper
C00-06	Lip, oral cavity	Lip, oral cavity
C07-08	Salivary glands	Salivary glands
C09-10	Oropharynx	Oropharynx
C11	Nasopharynx	Nasopharynx
C12-13	Hypopharynx	Hypopharynx
C15	Esophagus	Esophagus
C16	Stomach	Stomach
C18	Colon	Colon
C19-20	Rectum	Rectum
C18-21	Colorectum	Colorectum
C21	Anus	Anus
C22	Liver and intrahepatic bile ducts	Liver
C23	Gallbladder	Gallbladder
C25	Pancreas	Pancreas
C32	Larynx	Larynx
C33-34	Trachea, bronchus, and lung	Lung
C43	Melanoma of the skin	Melanoma of the skin
C44	Non-melanoma skin cancer	Skin
C45	Mesothelioma	Mesothelioma
C46	Kaposi sarcoma	Kaposi sarcoma
C50	Breast	Breast
C51	Vulva	Vulva
C52	Vagina	Vagina
C53	Cervix uteri	Cervix
C54	Corpus uteri	Corpus
C56	Ovary	Ovary
C60	Penis	Penis
C61	Prostate	Prostate
C62	Testis	Testis
C64	Kidney	Kidney
C67	Bladder	Bladder
C70-72	Brain, central nervous system	Brain, CNS
C73	Thyroid	Thyroid
C76-80, C96-97	Unspecified sites	Unspecified
C81	Hodgkin lymphoma	Hodgkin lymphoma
C82-86, C88	Non-Hodgkin lymphoma	Non-Hodgkin lymphoma
C90	Multiple myeloma	Multiple myeloma
C91-95	Leukemia	Leukemia
C17, C24, C30-31, C37-38, C40-41, C47-49, C57-58, C63, C65-66, C68-69, C74-75	Other specified cancers	Other sites
C00-97	All cancers	All sites
C00-97/C44	All cancers, excluding non-melanoma skin cancer	All sites, excluding skin

ICD-10 international statistical classification of diseases and related health problems, 10th revision

Table S2 The annual collection status of the mortality data by country, 2003 − 2022

Countries	2003	2004	2005	2006	2007	2008	2009	2010	2011	2012	2013	2014	2015	2016	2017	2018	2019	2020	2021	2022
Argentina	r	r	r	r	r	r	r	r	r	r	r	r	r	r	r	r	r	r	r	r
Australia	r	r	e	r	r	r	r	r	r	r	r	r	r	r	r	r	r	r	r	r
Austria	r	r	r	r	r	r	r	r	r	r	r	r	r	r	r	r	r	r	r	r
Belgium	r	r	r	r	r	r	r	r	r	r	r	r	r	r	r	r	r	r	r	e
Brazil	r	r	r	r	r	r	r	r	r	r	r	r	r	r	r	r	r	r	r	e
Bulgaria	e	e	r	r	r	r	r	r	r	r	r	r	r	r	r	r	r	r	r	r
Canada	r	r	r	r	r	r	r	r	r	r	r	r	r	r	r	r	r	r	r	r
Chile	r	r	r	r	r	r	r	r	r	r	r	r	r	r	r	r	r	r	r	e
China*	r	r	r	r	r	r	r	r	r	r	r	r	r	r	r	r	r	e	e	e
Colombia	r	r	r	r	r	r	r	r	r	r	r	r	r	r	r	r	r	r	r	e
Costa Rica	r	r	r	r	r	r	r	r	r	r	r	r	r	r	r	r	r	r	r	r
Croatia	r	r	r	r	r	r	r	r	r	r	r	r	r	r	r	r	r	r	r	e
Cuba	r	r	r	r	r	r	r	r	r	r	r	r	r	r	r	r	r	r	r	e
Cyprus	e	r	r	r	r	r	r	r	r	r	r	r	r	r	r	r	r	r	r	r
Czechia	r	r	r	r	r	r	r	r	r	r	r	r	r	r	r	r	r	r	r	r
Denmark	r	r	r	r	r	r	r	r	r	r	r	r	r	r	r	r	r	r	r	r
Ecuador	r	r	r	r	r	r	r	r	r	r	r	r	r	r	r	r	r	r	r	r
Estonia	r	r	r	r	r	r	r	r	r	r	r	r	r	r	r	r	r	r	r	r
Finland	r	r	r	r	r	r	r	r	r	r	r	r	r	r	r	r	r	r	r	r
France	r	r	r	r	r	r	r	r	r	r	r	r	r	r	r	r	r	r	r	r
Germany	r	r	r	r	r	r	r	r	r	r	r	r	r	r	r	r	r	r	e	e
Guatemala	e	e	r	r	r	r	r	r	r	r	r	r	r	r	r	r	r	r	r	r
Hungary	r	r	r	r	r	r	r	r	r	r	r	r	r	r	r	r	r	r	r	r
Iceland	r	r	r	r	r	r	r	r	r	r	r	r	r	r	r	r	r	r	r	r
Israel	r	r	r	r	r	r	r	r	r	r	r	r	r	r	r	r	r	r	r	r
Italy	r	r	r	r	r	r	r	r	r	r	r	r	r	r	r	r	r	r	r	e
Japan	r	r	r	r	r	r	r	r	r	r	r	r	r	r	r	r	r	r	r	e
Latvia	r	r	r	r	r	r	r	r	r	r	r	r	r	r	r	r	r	r	r	r

Countries	2003	2004	2005	2006	2007	2008	2009	2010	2011	2012	2013	2014	2015	2016	2017	2018	2019	2020	2021	2022
Lithuania	r	r	r	r	r	r	r	r	r	r	r	r	r	r	r	r	r	r	r	r
Luxembourg	r	r	r	r	r	r	r	r	r	r	r	r	r	r	r	r	r	r	r	r
Malta	r	r	r	r	r	r	r	r	r	r	r	r	r	r	r	r	r	r	r	e
Mauritius	e	e	r	r	r	r	r	r	r	r	r	r	r	r	r	r	r	r	r	r
Mexico	r	r	r	r	r	r	r	r	r	r	r	r	r	r	r	r	r	r	r	r
Netherlands	r	r	r	r	r	r	r	r	r	r	r	r	r	r	r	r	r	r	r	r
Nicaragua	r	r	r	r	r	r	r	r	r	r	r	r	r	r	r	r	r	r	r	r
Norway	r	r	r	r	r	r	r	r	r	r	r	r	r	r	r	r	r	r	r	r
Paraguay	r	r	r	r	r	r	r	r	r	r	r	r	r	r	r	r	r	r	r	e
Peru	r	r	r	r	r	r	r	r	r	r	r	r	r	r	r	r	r	r	e	e
Poland	r	r	r	r	r	r	r	r	r	r	r	r	r	r	r	r	r	r	r	r
Republic of Korea	r	r	r	r	r	r	r	r	r	r	r	r	r	r	r	r	r	r	r	r
Serbia	r	r	r	r	r	r	r	r	r	r	r	r	r	r	r	r	r	r	r	r
Slovenia	r	r	r	r	r	r	r	r	r	r	r	r	r	r	r	r	r	r	e	e
Spain	r	r	r	r	r	r	r	r	r	r	r	r	r	r	r	r	r	r	r	r
Sweden	r	r	r	r	r	r	r	r	r	r	r	r	r	r	r	r	r	r	r	r
Switzerland	r	r	r	r	r	r	r	r	r	r	r	r	r	r	r	r	r	r	r	r
United Kingdom	r	r	r	r	r	r	r	r	r	r	r	r	r	r	r	r	r	r	r	e
United States of America	r	r	r	r	r	r	r	r	r	r	r	r	r	r	r	r	r	r	r	r

*The mortality data of China were extracted from the China Cancer Registry Annual Reports. “r” denoted reported data collected from the World Health Organization mortality database or cancer registry annual reports. “e” denoted estimated data based on the trends observed in the nearest 10-year window

Table S3 Data intervals for estimating missing mortality data in specified years, by country

**Countries**	**Missing year**	**Data employed for the estimation of the missing values from the specified year**
Australia	2005	2003 − 2012
Belgium	2022	2012 − 2021
Brazil	2022	2012 − 2021
Bulgaria	2003, 2004	2005 − 2014
Chile	2022	2012 − 2021
China	2019, 2020, 2021	2010 − 2019
Colombia	2022	2012 − 2021
Croatia	2022	2012 − 2021
Cuba	2022	2012 − 2021
Cyprus	2003	2004 − 2013
Germany	2021, 2022	2011 − 2020
Guatemala	2003, 2004	2005 − 2014
Italy	2022	2012 − 2021
Japan	2022	2012 − 2021
Malta	2022	2012 − 2021
Mauritius	2003, 2004	2005 − 2014
Paraguay	2022	2012 − 2021
Peru	2021, 2022	2011 − 2020
Slovenia	2021, 2022	2011 − 2020
United Kingdom	2022	2012 − 2021

Table S4 The step-by-step calculation of the life expectancy

***x* (year)**	***n***	**P_x (×10^5)**	**D_x**	**m_x (×10^{-4})**	**q_x (×10^{-4})**	**p_x (%)**	**l_x (×10^4)**	**d_x**	**L_x (×10^4)**	**T_x (×10^6)**	**e_x**
0	1	1.26	1129	89.60	89.60	99.10	10.00	896.03	9.92	8.10	80.96
1	4	4.26	421	9.88	39.45	99.61	9.91	390.99	39.56	8.00	80.69
5	5	6.21	153	2.46	12.31	99.88	9.87	121.53	49.33	7.60	77.01
10	5	6.23	185	2.97	14.84	99.85	9.86	146.28	49.26	7.11	72.10
15	5	5.36	186	3.47	17.34	99.83	9.84	170.66	49.18	6.62	67.20
20	5	5.02	299	5.96	29.74	99.70	9.83	292.23	49.06	6.12	62.31
25	5	5.80	365	6.29	31.42	99.69	9.80	307.82	48.91	5.63	57.49
30	5	7.99	548	6.86	34.23	99.66	9.77	334.38	48.75	5.14	52.67
35	5	7.42	528	7.12	35.52	99.64	9.73	345.72	48.58	4.66	47.84
40	5	6.68	715	10.70	53.38	99.47	9.70	517.71	48.37	4.17	43.00
45	5	7.15	1064	14.88	74.13	99.26	9.65	715.18	48.06	3.69	38.22
50	5	8.65	2010	23.24	115.51	98.84	9.58	1106.18	47.60	3.21	33.48
55	5	8.00	2917	36.46	180.67	98.19	9.47	1710.10	46.90	2.73	28.85
60	5	4.80	3032	63.17	310.92	96.89	9.29	2889.88	45.75	2.26	24.33
65	5	5.41	5541	102.42	499.32	95.01	9.01	4496.66	43.90	1.80	20.03
70	5	3.93	7535	191.73	914.80	90.85	8.56	7826.93	40.82	1.36	15.95
75	5	2.40	8160	340.00	1566.82	84.34	7.77	12,179.16	35.82	0.96	12.31
80	5	1.51	9857	652.78	2805.98	71.86	6.56	18,393.94	28.18	0.60	9.13
≥ 85	5	1.22	18,172	1489.51	10,000.00	0.00	4.72	47,158.62	31.66	0.32	6.71

x referred to age group, n referred to age interval, P_x referred to average population, D_x referred to number of all cause deaths, m_x referred to all-cause mortality rate ate age x, q_x referred to the probability of dying at age x, p_x referred to the probability of surviving at age x, l_x referred to the number of people alive at age x, d_x referred to the number of deaths in the interval (x, $x+n$), L_x referred to the total number of person-years lived from age x to $x+n$, T_x referred to the total number of person-years lived from age x until ≥ 85, e_x referred to the life expectancy at age x

Table S5 AAPCs for premature cancer death indicators by country, 2003 − 2022

Country	Probability of premature cancer-related death		The ASR of YLLs caused by cancer-related premature death		The PGLEs resulting from the elimination of premature cancer death	
	AAPC [% (95% CI)]	*P*-value	AAPC [% (95% CI)]	*P*-value	AAPC [% (95% CI)]	*P*-value
Mauritius	-0.04 (-0.45 to 0.37)	0.828	0.08 (-0.29 to 0.46)	0.645	0.69 (0.19 − 1.18)	0.009
China	-1.63 (-1.81 to -1.45)	< 0.001	-2.05 (-2.25 to -1.85)	< 0.001	-1.09 (-1.41 to -0.77)	< 0.001
Japan	-1.89 (-2.01 to -1.77)	< 0.001	-2.16 (-2.28 to -2.05)	< 0.001	-1.63 (-1.87 to -1.39)	< 0.001
Republic of Korea	-3.66 (-3.72 to -3.60)	< 0.001	-3.77 (-3.84 to -3.71)	< 0.001	-2.75 (-3.17 to -2.32)	< 0.001
Cyprus	-0.35 (-0.78 to 0.09)	0.110	-0.05 (-0.55 to 0.45)	0.826	-0.43 (-2.05 to 1.21)	0.603
Israel	-2.10 (-2.44 to -1.77)	< 0.001	-2.27 (-2.59 to -1.96)	< 0.001	-1.82 (-2.33 to -1.31)	< 0.001
Bulgaria	-1.30 (-1.96 to -0.64)	< 0.001	-1.38 (-1.61 to -1.16)	< 0.001	-1.78 (-2.72 to -0.82)	< 0.001
Czechia	-2.52 (-2.62 to -2.41)	< 0.001	-2.95 (-3.28 to -2.63)	< 0.001	-2.46 (-2.89 to -2.02)	< 0.001
Hungary	-1.45 (-1.76 to -1.13)	< 0.001	-2.21 (-2.48 to -1.94)	< 0.001	-1.63 (-2.20 to -1.07)	< 0.001
Poland	-1.87 (-2.12 to -1.63)	< 0.001	-2.29 (-2.56 to -2.03)	< 0.001	-1.92 (-2.45 to -1.38)	< 0.001
Denmark	-2.45 (-2.78 to -2.13)	< 0.001	-2.82 (-3.12 to -2.53)	< 0.001	-1.86 (-2.23 to -1.49)	< 0.001
Estonia	-2.10 (-2.84 to -1.36)	< 0.001	-2.19 (-2.47 to -1.9)	< 0.001	-0.86 (-1.44 to -0.27)	0.004
Finland	-1.24 (-1.46 to -1.01)	< 0.001	-1.50 (-1.65 to -1.36)	< 0.001	-0.87 (-1.24 to -0.50)	< 0.001
Iceland	-1.93 (-2.50 to -1.36)	< 0.001	-1.92 (-2.40 to -1.43)	< 0.001	-1.56 (-2.10 to -1.02)	< 0.001
Latvia	-1.02 (-1.20 to -0.85)	< 0.001	-1.30 (-1.50 to -1.1)	< 0.001	-0.61 (-1.42 to 0.21)	0.143
Lithuania	-0.97 (-1.45 to -0.48)	< 0.001	-1.39 (-1.63 to -1.15)	< 0.001	-0.76 (-1.51 to -0.01)	0.048
Netherlands	-1.80 (-2.05 to -1.56)	< 0.001	-2.03 (-2.28 to -1.79)	< 0.001	-1.55 (-1.88 to -1.22)	< 0.001
Sweden	-2.30 (-2.68 to -1.91)	< 0.001	-2.59 (-2.87 to -2.32)	< 0.001	-2.01 (-2.57 to -1.45)	< 0.001
United Kingdom	-1.68 (-1.77 to -1.60)	< 0.001	-1.71 (-1.79 to -1.63)	< 0.001	-1.34 (-1.68 to -1.00)	< 0.001
Austria	-1.74 (-1.92 to -1.56)	< 0.001	-2.16 (-2.36 to -1.96)	< 0.001	-1.54 (-1.89 to -1.19)	< 0.001
Croatia	-0.93 (-1.23 to -0.63)	< 0.001	-1.22 (-1.51 to -0.93)	< 0.001	-1.06 (-1.71 to -0.41)	0.001
Italy	-1.91 (-2.16 to -1.66)	< 0.001	-2.01 (-2.26 to -1.75)	< 0.001	-1.55 (-1.87 to -1.22)	< 0.001
Malta	-1.79 (-2.24 to -1.35)	< 0.001	-2.37 (-3.32 to -1.40)	< 0.001	-1.45 (-2.53 to -0.36)	0.009
Serbia	-0.78 (-1.17 to -0.39)	< 0.001	-1.23 (-1.59 to -0.87)	< 0.001	-1.07 (-1.85 to -0.28)	0.008
Slovenia	-1.47 (-1.68 to -1.25)	< 0.001	-1.95 (-2.15 to -1.75)	< 0.001	-1.10 (-1.65 to -0.56)	< 0.001

Country	Probability of premature cancer-related death		The ASR of YLLs caused by cancer-related premature death		The PGLEs resulting from the elimination of premature cancer death	
	AAPC [% (95% CI)]	*P*-value	AAPC [% (95% CI)]	*P*-value	AAPC [% (95% CI)]	*P*-value
Spain	-1.59 (-1.80 to -1.37)	< 0.001	-1.99 (-2.12 to -1.85)	< 0.001	-1.19 (-1.51 to -0.86)	< 0.001
Belgium	-2.12 (-2.34 to -1.91)	< 0.001	-2.51 (-2.73 to -2.29)	< 0.001	-1.89 (-2.28 to -1.50)	< 0.001
France	-1.79 (-1.97 to -1.60)	< 0.001	-2.20 (-2.54 to -1.86)	< 0.001	-1.64 (-1.89 to -1.38)	< 0.001
Germany	-1.29 (-1.43 to -1.14)	< 0.001	-1.61 (-1.74 to -1.48)	< 0.001	-1.32 (-1.57 to -1.07)	< 0.001
Luxembourg	-2.52 (-3.38 to -1.65)	< 0.001	-2.94 (-3.68 to -2.20)	< 0.001	-1.98 (-2.74 to -1.23)	< 0.001
Norway	-2.38 (-2.65 to -2.12)	< 0.001	-2.59 (-2.85 to -2.32)	< 0.001	-2.04 (-2.29 to -1.80)	< 0.001
Switzerland	-2.28 (-2.54 to -2.03)	< 0.001	-2.45 (-2.71 to -2.19)	< 0.001	-1.57 (-2.12 to -1.02)	< 0.001
Cuba	-0.14 (-0.32 to 0.04)	0.136	-0.45 (-0.63 to -0.26)	< 0.001	-1.95 (-2.57 to -1.32)	< 0.001
Costa Rica	-0.02 (-0.44 to 0.41)	0.942	-0.26 (-1.00 to 0.49)	0.501	0.23 (-0.11 to 0.57)	0.170
Guatemala	-1.54 (-1.75 to -1.33)	< 0.001	-1.71 (-1.91 to -1.52)	< 0.001	-1.76 (-2.25 to -1.27)	< 0.001
Mexico	-0.88 (-1.15 to -0.61)	< 0.001	-0.81 (-1.10 to -0.53)	< 0.001	-1.49 (-1.99 to -0.98)	< 0.001
Nicaragua	0.12 (-0.56 to 0.81)	0.728	-0.10 (-0.80 to 0.62)	0.791	-0.73 (-1.36 to -0.10)	0.024
Argentina	-1.30 (-1.50 to -1.09)	< 0.001	-1.30 (-1.49 to -1.12)	< 0.001	-1.35 (-1.88 to -0.82)	< 0.001
Brazil	-0.69 (-0.88 to -0.49)	< 0.001	-0.62 (-0.82 to -0.42)	< 0.001	-0.91 (-1.23 to -0.59)	< 0.001
Chile	-1.60 (-1.82 to -1.37)	< 0.001	-1.76 (-2.26 to -1.26)	< 0.001	-1.40 (-1.92 to -0.88)	< 0.001
Colombia	-1.76 (-2.02 to -1.49)	< 0.001	-1.54 (-1.76 to -1.32)	< 0.001	-1.94 (-2.38 to -1.50)	< 0.001
Ecuador	-0.50 (-0.71 to -0.29)	< 0.001	-0.15 (-0.90 to 0.60)	0.686	-0.47 (-0.94 to 0.00)	0.048
Paraguay	0.25 (0.04 – 0.47)	0.021	0.13 (-0.04 to 0.31)	0.127	-1.75 (-2.49 to -1.01)	< 0.001
Peru	0.32 (-0.41 to 1.05)	0.391	0.33 (-0.43 to 1.09)	0.395	-0.48 (-1.78 to 0.83)	0.470
Canada	-2.18 (-2.31 to -2.06)	< 0.001	-2.34 (-2.50 to -2.18)	< 0.001	-2.02 (-2.17 to -1.87)	< 0.001
United States of America	-1.89 (-2.05 to -1.73)	< 0.001	-1.97 (-2.13 to -1.81)	< 0.001	-2.17 (-2.50 to -1.85)	< 0.001
Australia	-1.78 (-1.88 to -1.68)	< 0.001	-1.83 (-1.95 to -1.70)	< 0.001	-1.57 (-1.84 to -1.30)	< 0.001

AAPC average annual percent change, *ASR* age-standardized rate, *CI* confidence interval, *YLLs* years of life lost, *PGLEs* potential gains in life expectancy

Table S6 AAPCs for premature cancer death indicators by country among men, 2003 − 2022

Country	Probability of premature cancer-related death		The ASR of YLLs caused by cancer-related premature death		The PGLEs resulting from the elimination of premature cancer death	
	AAPC [% (95% CI)]	*P*-value	AAPC [% (95% CI)]	*P*-value	AAPC [% (95% CI)]	*P*-value
Mauritius	-0.29 (-0.98 to 0.40)	0.382	-0.20 (-0.85 to 0.44)	0.516	0.61 (-0.24 to 1.46)	0.148
China	-1.39 (-1.62 to -1.15)	< 0.001	-1.98 (-2.23 to -1.74)	< 0.001	-1.03 (-1.37 to -0.68)	< 0.001
Japan	-2.41 (-2.53 to -2.29)	< 0.001	-2.81 (-2.92 to -2.7)	< 0.001	-2.05 (-2.23 to -1.86)	< 0.001
Republic of Korea	-4.23 (-4.30 to -4.16)	< 0.001	-4.54 (-4.61 to -4.47)	< 0.001	-3.19 (-3.63 to -2.74)	< 0.001
Cyprus	-0.31 (-0.97 to 0.36)	0.342	-0.03 (-0.77 to 0.72)	0.940	-0.49 (-3.17 to 2.26)	0.723
Israel	-1.97 (-2.30 to -1.63)	< 0.001	-2.14 (-2.49 to -1.79)	< 0.001	-1.57 (-2.03 to -1.1)	< 0.001
Bulgaria	-1.76 (-2.48 to -1.03)	< 0.001	-2.17 (-3.02 to -1.31)	< 0.001	-2.45 (-3.54 to -1.35)	< 0.001
Czechia	-2.96 (-3.09 to -2.83)	< 0.001	-3.46 (-4.12 to -2.80)	< 0.001	-2.91 (-3.52 to -2.29)	< 0.001
Hungary	-2.07 (-2.45 to -1.69)	< 0.001	-2.91 (-3.32 to -2.49)	< 0.001	-2.13 (-2.86 to -1.38)	< 0.001
Poland	-2.44 (-2.77 to -2.10)	< 0.001	-2.69 (-2.84 to -2.55)	< 0.001	-2.38 (-2.99 to -1.76)	< 0.001
Denmark	-2.46 (-2.82 to -2.10)	< 0.001	-2.86 (-3.20 to -2.52)	< 0.001	-1.71 (-2.09 to -1.33)	< 0.001
Estonia	-2.42 (-3.28 to -1.55)	< 0.001	-2.50 (-2.84 to -2.16)	< 0.001	-0.77 (-1.44 to -0.09)	0.026
Finland	-1.45 (-1.61 to -1.28)	< 0.001	-1.61 (-1.79 to -1.44)	< 0.001	-0.65 (-1.12 to -0.18)	0.007
Iceland	-0.64 (-3.28 to 2.06)	0.638	-1.93 (-2.82 to -1.03)	< 0.001	-0.27 (-3.23 to 2.79)	0.862
Latvia	-1.30 (-1.48 to -1.12)	< 0.001	-1.62 (-1.82 to -1.42)	< 0.001	-0.19 (-0.91 to 0.54)	0.609
Lithuania	-1.32 (-2.17 to -0.45)	0.003	-1.37 (-2.01 to -0.73)	< 0.001	-0.58 (-1.37 to 0.22)	0.155
Netherlands	-2.17 (-2.44 to -1.91)	< 0.001	-2.33 (-2.61 to -2.06)	< 0.001	-1.61 (-2.05 to -1.16)	< 0.001
Sweden	-2.54 (-2.88 to -2.20)	< 0.001	-2.57 (-2.82 to -2.32)	< 0.001	-1.84 (-2.30 to -1.38)	< 0.001
United Kingdom	-1.76 (-1.84 to -1.67)	< 0.001	-1.77 (-1.85 to -1.69)	< 0.001	-1.32 (-1.71 to -0.92)	< 0.001
Austria	-2.02 (-2.25 to -1.79)	< 0.001	-2.47 (-2.71 to -2.23)	< 0.001	-1.73 (-2.09 to -1.37)	< 0.001
Croatia	-1.51 (-1.80 to -1.22)	< 0.001	-1.91 (-2.28 to -1.54)	< 0.001	-1.72 (-2.19 to -1.25)	< 0.001
Italy	-2.45 (-2.61 to -2.29)	< 0.001	-2.54 (-2.71 to -2.37)	< 0.001	-2.08 (-2.45 to -1.71)	< 0.001
Malta	-1.76 (-2.37 to -1.15)	< 0.001	-2.15 (-3.61 to -0.67)	0.005	-1.04 (-2.57 to 0.50)	0.185
Serbia	-1.17 (-1.62 to -0.71)	< 0.001	-1.68 (-2.15 to -1.20)	< 0.001	-1.62 (-2.63 to -0.60)	0.002
Slovenia	-1.81 (-2.02 to -1.60)	< 0.001	-2.25 (-2.64 to -1.85)	< 0.001	-0.94 (-1.47 to -0.40)	0.001
Spain	-2.11 (-2.28 to -1.94)	< 0.001	-2.67 (-2.82 to -2.52)	< 0.001	-1.71 (-2.04 to -1.38)	< 0.001

Country	Probability of premature cancer-related death		The ASR of YLLs caused by cancer-related premature death		The PGLEs resulting from the elimination of premature cancer death	
	AAPC [% (95% CI)]	*P*-value	AAPC [% (95% CI)]	*P*-value	AAPC [% (95% CI)]	*P*-value
Belgium	-2.6 (-2.89 to -2.31)	< 0.001	-3.07 (-3.40 to -2.74)	< 0.001	-2.25 (-2.69 to -1.80)	< 0.001
France	-2.34 (-2.52 to -2.15)	< 0.001	-2.81 (-2.98 to -2.63)	< 0.001	-2.08 (-2.33 to -1.83)	< 0.001
Germany	-1.55 (-1.68 to -1.41)	< 0.001	-1.95 (-2.10 to -1.80)	< 0.001	-1.57 (-1.75 to -1.38)	< 0.001
Luxembourg	-3.06 (-3.64 to -2.48)	< 0.001	-3.44 (-4.05 to -2.82)	< 0.001	-2.33 (-3.02 to -1.64)	< 0.001
Norway	-2.59 (-2.94 to -2.23)	< 0.001	-2.64 (-3.42 to -1.85)	< 0.001	-2.02 (-2.33 to -1.70)	< 0.001
Switzerland	-2.64 (-3.01 to -2.26)	< 0.001	-2.83 (-3.05 to -2.60)	< 0.001	-1.73 (-2.47 to -0.99)	< 0.001
Cuba	-0.03 (-0.22 to 0.16)	0.765	-0.37 (-0.59 to -0.15)	0.001	-2.24 (-3.03 to -1.45)	< 0.001
Costa Rica	-0.13 (-0.68 to 0.43)	0.643	-0.20 (-0.74 to 0.35)	0.458	0.06 (-0.41 to 0.53)	0.792
Guatemala	-1.38 (-1.69 to -1.06)	< 0.001	-1.74 (-2.04 to -1.45)	< 0.001	-2.03 (-2.81 to -1.24)	< 0.001
Mexico	-0.92 (-1.31 to -0.53)	< 0.001	-0.79 (-1.22 to -0.35)	< 0.001	-1.79 (-2.48 to -1.10)	< 0.001
Nicaragua	0.57 (-0.24 to 1.39)	0.171	0.31 (-0.14 to 0.75)	0.163	-0.58 (-1.44 to 0.29)	0.190
Argentina	-2.05 (-2.30 to -1.80)	< 0.001	-2.11 (-2.34 to -1.88)	< 0.001	-2.08 (-2.82 to -1.32)	< 0.001
Brazil	-1.03 (-1.22 to -0.84)	< 0.001	-1.06 (-1.34 to -0.77)	< 0.001	-1.37 (-1.71 to -1.02)	< 0.001
Chile	-1.58 (-2.28 to -0.88)	< 0.001	-1.75 (-2.08 to -1.43)	< 0.001	-1.16 (-1.60 to -0.72)	< 0.001
Colombia	-1.99 (-2.27 to -1.71)	< 0.001	-1.81 (-2.03 to -1.59)	< 0.001	-2.23 (-2.65 to -1.81)	< 0.001
Ecuador	-0.65 (-0.87 to -0.42)	< 0.001	-0.6 (-0.83 to -0.37)	< 0.001	-0.56 (-1.16 to 0.06)	0.073
Paraguay	0.58 (0.25 – 0.92)	0.002	0.38 (0.09 – 0.67)	0.014	-2.02 (-3.09 to -0.94)	< 0.001
Peru	0.35 (-0.43 to 1.14)	0.380	0.40 (-0.40 to 1.20)	0.330	-1.66 (-3.18 to -0.11)	0.036
Canada	-2.33 (-2.40 to -2.25)	< 0.001	-2.56 (-2.78 to -2.35)	< 0.001	-2.14 (-2.35 to -1.92)	< 0.001
United States of America	-2.18 (-2.42 to -1.95)	< 0.001	-2.32 (-2.50 to -2.14)	< 0.001	-2.46 (-2.84 to -2.09)	< 0.001
Australia	-1.84 (-1.96 to -1.71)	< 0.001	-1.87 (-2.02 to -1.71)	< 0.001	-1.49 (-1.82 to -1.15)	< 0.001

AAPC average annual percent change, *ASR* age-standardized rate, *CI* confidence interval, *YLLs* years of life lost, *PGLEs* potential gains in life expectancy

Table S7 AAPCs for premature cancer death indicators by country among women, 2003 − 2022

Country	Probability of premature cancer-related death		The ASR of YLLs caused by cancer-related premature death		The PGLEs resulting from the elimination of premature cancer death	
	AAPC [% (95% CI)]	*P*-value	AAPC [% (95% CI)]	*P*-value	AAPC [% (95% CI)]	*P*-value
Mauritius	0.17 (-0.22 to 0.56)	0.376	0.25 (-0.34 to 0.84)	0.415	0.78 (0.41 − 1.15)	< 0.001
China	-2.09 (-2.29 to -1.88)	< 0.001	-2.16 (-2.37 to -1.94)	< 0.001	-1.33 (-1.56 to -1.10)	< 0.001
Japan	-1.05 (-1.21 to -0.89)	< 0.001	-1.28 (-1.44 to -1.13)	< 0.001	-0.91 (-1.23 to -0.59)	< 0.001
Republic of Korea	-2.64 (-2.90 to -2.37)	< 0.001	-2.58 (-2.71 to -2.44)	< 0.001	-1.70 (-1.87 to -1.54)	< 0.001
Cyprus	-0.42 (-0.91 to 0.08)	0.093	-0.63 (-2.96 to 1.76)	0.604	-0.34 (-1.92 to 1.26)	0.673
Israel	-2.24 (-2.68 to -1.80)	< 0.001	-2.42 (-2.85 to -1.99)	< 0.001	-2.33 (-3.02 to -1.63)	< 0.001
Bulgaria	-0.52 (-0.75 to -0.30)	< 0.001	-0.86 (-1.11 to -0.61)	< 0.001	-0.63 (-1.02 to -0.25)	0.003
Czechia	-1.95 (-2.07 to -1.83)	< 0.001	-2.21 (-2.36 to -2.05)	< 0.001	-1.87 (-2.29 to -1.46)	< 0.001
Hungary	-0.77 (-1.33 to -0.20)	0.008	-1.30 (-1.69 to -0.91)	< 0.001	-0.75 (-1.20 to -0.30)	0.001
Poland	-1.22 (-1.50 to -0.94)	< 0.001	-1.72 (-2.00 to -1.44)	< 0.001	-1.20 (-1.70 to -0.70)	< 0.001
Denmark	-2.39 (-2.59 to -2.19)	< 0.001	-2.70 (-2.90 to -2.50)	< 0.001	-1.94 (-2.17 to -1.71)	< 0.001
Estonia	-1.77 (-2.42 to -1.10)	< 0.001	-1.94 (-2.27 to -1.61)	< 0.001	-0.95 (-1.75 to -0.13)	0.022
Finland	-1.05 (-1.37 to -0.73)	< 0.001	-1.40 (-1.59 to -1.22)	< 0.001	-0.93 (-1.33 to -0.52)	< 0.001
Iceland	-2.03 (-2.74 to -1.31)	< 0.001	-1.87 (-2.55 to -1.19)	< 0.001	-1.72 (-2.42 to -1.02)	< 0.001
Latvia	-0.77 (-1.14 to -0.40)	< 0.001	-1.01 (-1.41 to -0.61)	< 0.001	-0.63 (-1.73 to 0.48)	0.263
Lithuania	-0.85 (-1.14 to -0.57)	< 0.001	-1.20 (-1.51 to -0.89)	< 0.001	-0.56 (-1.00 to -0.12)	0.016
Netherlands	-1.35 (-1.64 to -1.07)	< 0.001	-1.77 (-2.05 to -1.49)	< 0.001	-1.34 (-1.61 to -1.07)	< 0.001
Sweden	-2.40 (-2.76 to -2.03)	< 0.001	-2.62 (-3.00 to -2.24)	< 0.001	-2.33 (-2.76 to -1.89)	< 0.001
United Kingdom	-1.69 (-1.88 to -1.50)	< 0.001	-1.64 (-1.75 to -1.54)	< 0.001	-1.32 (-1.67 to -0.97)	< 0.001
Austria	-1.37 (-1.60 to -1.150)	< 0.001	-1.72 (-1.99 to -1.45)	< 0.001	-1.19 (-1.58 to -0.8)	< 0.001
Croatia	-0.02 (-0.39 to 0.34)	0.898	-0.35 (-0.64 to -0.06)	0.021	0.01 (-0.50 to 0.52)	0.971
Italy	-1.03 (-1.12 to -0.93)	< 0.001	-1.19 (-1.29 to -1.09)	< 0.001	-0.93 (-1.27 to -0.58)	< 0.001
Malta	-1.93 (-2.33 to -1.53)	< 0.001	-2.04 (-2.64 to -1.44)	< 0.001	-1.37 (-1.93 to -0.81)	< 0.001
Serbia	-0.23 (-0.40 to -0.06)	0.012	-0.77 (-1.09 to -0.45)	< 0.001	-0.41 (-1.00 to 0.19)	0.176
Slovenia	-1.41 (-2.07 to -0.74)	< 0.001	-1.33 (-1.66 to -1.00)	< 0.001	-1.18 (-1.79 to -0.56)	< 0.001

Country	Probability of premature cancer-related death		The ASR of YLLs caused by cancer-related premature death		The PGLEs resulting from the elimination of premature cancer death	
	AAPC [% (95% CI)]	*P*-value	AAPC [% (95% CI)]	*P*-value	AAPC [% (95% CI)]	*P*-value
Spain	-0.54 (-0.86 to -0.21)	0.001	-0.91 (-1.10 to -0.72)	< 0.001	-0.07 (-0.76 to 0.62)	0.845
Belgium	-1.48 (-1.67 to -1.30)	< 0.001	-1.88 (-2.10 to -1.66)	< 0.001	-1.40 (-1.67 to -1.12)	< 0.001
France	-0.92 (-1.30 to -0.54)	< 0.001	-1.21 (-1.64 to -0.78)	< 0.001	-0.74 (-1.01 to -0.48)	< 0.001
Germany	-0.89 (-1.06 to -0.71)	< 0.001	-1.16 (-1.34 to -0.97)	< 0.001	-1.00 (-1.29 to -0.72)	< 0.001
Luxembourg	-1.59 (-3.07 to -0.10)	0.037	-2.16 (-3.48 to -0.83)	0.001	-1.47 (-2.63 to -0.30)	0.014
Norway	-2.19 (-2.55 to -1.83)	< 0.001	-2.52 (-2.69 to -2.34)	< 0.001	-2.14 (-2.51 to -1.78)	< 0.001
Switzerland	-1.83 (-2.18 to -1.47)	< 0.001	-2.05 (-2.41 to -1.68)	< 0.001	-1.36 (-1.88 to -0.84)	< 0.001
Cuba	-0.17 (-0.35 to 0.00)	0.054	-0.64 (-0.79 to -0.49)	< 0.001	-1.67 (-2.42 to -0.91)	< 0.001
Costa Rica	0.12 (-0.30 to 0.54)	0.548	0.19 (-0.20 to 0.58)	0.322	0.42 (0.08 to 0.76)	0.019
Guatemala	-1.70 (-1.95 to -1.45)	< 0.001	-1.37 (-1.92 to -0.82)	< 0.001	-1.89 (-2.32 to -1.45)	< 0.001
Mexico	-0.84 (-1.28 to -0.40)	< 0.001	-0.89 (-1.21 to -0.58)	< 0.001	-1.26 (-1.63 to -0.89)	< 0.001
Nicaragua	-0.06 (-0.52 to 0.40)	0.798	-0.29 (-1.12 to 0.54)	0.494	-0.79 (-1.47 to -0.10)	0.025
Argentina	-0.46 (-0.69 to -0.22)	< 0.001	-0.46 (-0.71 to -0.21)	< 0.001	-0.64 (-1.03 to -0.25)	0.001
Brazil	-0.17 (-0.35 to 0.02)	0.078	-0.10 (-0.31 to 0.11)	0.343	-0.40 (-0.71 to -0.10)	0.010
Chile	-1.55 (-1.69 to -1.41)	< 0.001	-1.60 (-1.74 to -1.46)	< 0.001	-1.48 (-1.92 to -1.04)	< 0.001
Colombia	-1.58 (-1.96 to -1.21)	< 0.001	-1.33 (-1.77 to -0.89)	< 0.001	-1.71 (-2.28 to -1.13)	< 0.001
Ecuador	-0.39 (-0.66 to -0.12)	0.008	-0.35 (-0.61 to -0.10)	0.010	-0.39 (-0.77 to -0.02)	0.040
Paraguay	0.26 (-0.31 to 0.83)	0.379	-0.05 (-0.45 to 0.35)	0.804	-1.16 (-2.09 to -0.22)	0.016
Peru	0.40 (-0.41 to 1.23)	0.334	0.39 (-0.48 to 1.27)	0.376	0.40 (-1.22 to 2.04)	0.633
Canada	-1.88 (-2.33 to -1.44)	< 0.001	-2.05 (-2.57 to -1.53)	< 0.001	-1.85 (-2.06 to -1.64)	< 0.001
United States of America	-1.58 (-1.79 to -1.36)	< 0.001	-1.66 (-1.71 to -1.61)	< 0.001	-1.84 (-2.13 to -1.55)	< 0.001
Australia	-1.65 (-1.74 to -1.56)	< 0.001	-1.74 (-1.86 to -1.61)	< 0.001	-1.49 (-1.60 to -1.37)	< 0.001

AAPC average annual percent change, *ASR* age-standardized rate, *CI* confidence interval, *YLLs* years of life lost, *PGLEs* potential gains in life expectancy

Table S8 Reduction proportion in probability of premature cancer-related death (2015 – 2022) by country and sex

Country	Both sexes (%)	Men (%)	Women (%)
Mauritius	3.68	11.20	-3.65
China	**16.77**	15.30	**18.35**
Japan	15.31	**19.50**	8.78
Republic of Korea	**22.54**	**25.71**	**16.35**
Cyprus	14.33	**17.72**	10.14
Israel	**17.70**	**17.79**	**17.56**
Bulgaria	15.36	**19.84**	8.27
Czechia	**16.46**	**18.23**	14.30
Hungary	15.42	**18.36**	11.07
Poland	**17.18**	**19.62**	13.97
Denmark	**19.74**	**21.31**	**17.97**
Estonia	**20.27**	**22.97**	**17.99**
Finland	11.90	14.36	8.78
Iceland	7.20	2.80	10.55
Latvia	9.45	7.55	13.96
Lithuania	15.42	**19.16**	11.93
Netherlands	**17.69**	**18.55**	**16.70**
Sweden	**17.75**	**17.51**	**17.96**
United Kingdom	12.57	12.00	12.75
Austria	15.31	**16.56**	13.94
Croatia	9.72	13.92	3.01
Italy	13.90	**18.18**	7.82
Malta	**21.93**	**25.26**	**17.47**
Serbia	12.88	**19.52**	3.03
Slovenia	14.54	12.83	**18.74**
Spain	12.34	**16.77**	4.21
Belgium	**22.66**	**25.48**	**18.99**
France	**15.75**	**19.87**	8.55
Germany	11.04	12.54	8.45
Luxembourg	**19.73**	**16.81**	**23.29**
Norway	**15.96**	**17.76**	13.92
Switzerland	**19.59**	**22.39**	**16.13**
Cuba	6.10	8.55	1.99
Costa Rica	2.74	5.48	-0.58
Guatemala	11.57	12.41	11.00
Mexico	6.36	6.21	6.57
Nicaragua	8.71	9.25	8.40
Argentina	13.85	**19.53**	7.41
Brazil	8.70	11.35	5.59
Chile	12.93	13.78	11.88
Colombia	7.99	11.02	5.07
Ecuador	1.01	2.54	-0.31
Paraguay	-0.80	4.35	-8.02
Peru	-20.43	-18.44	-21.85

Country	Both sexes (%)	Men (%)	Women (%)
Canada	**15.67**	**16.74**	14.48
United States of America	13.28	**15.93**	10.21
Australia	12.51	12.16	12.45

Values in bold indicate reductions exceeding 15.50%

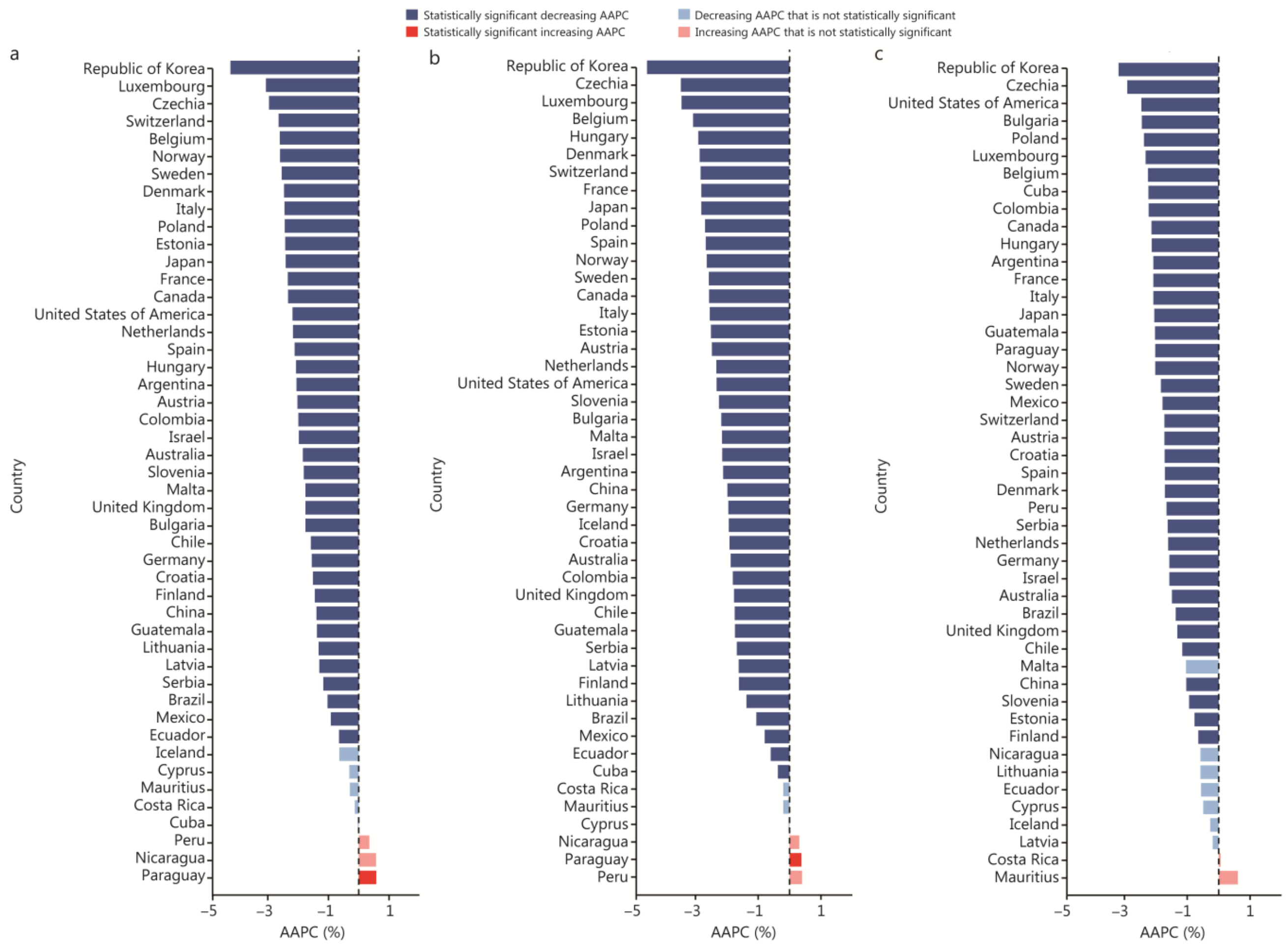

Fig. S1 AAPCs for premature cancer death indicators by country among men, 2003 − 2022. **a** Probability of premature cancer-related death. **b** The ASR of YLLs caused by cancer-related premature death. **c** The PGLEs resulting from the elimination of premature cancer death. AAPC average annual percent change, ASR age-standardized rate, YLLs years of life lost, PGLEs potential gains in life expectancy

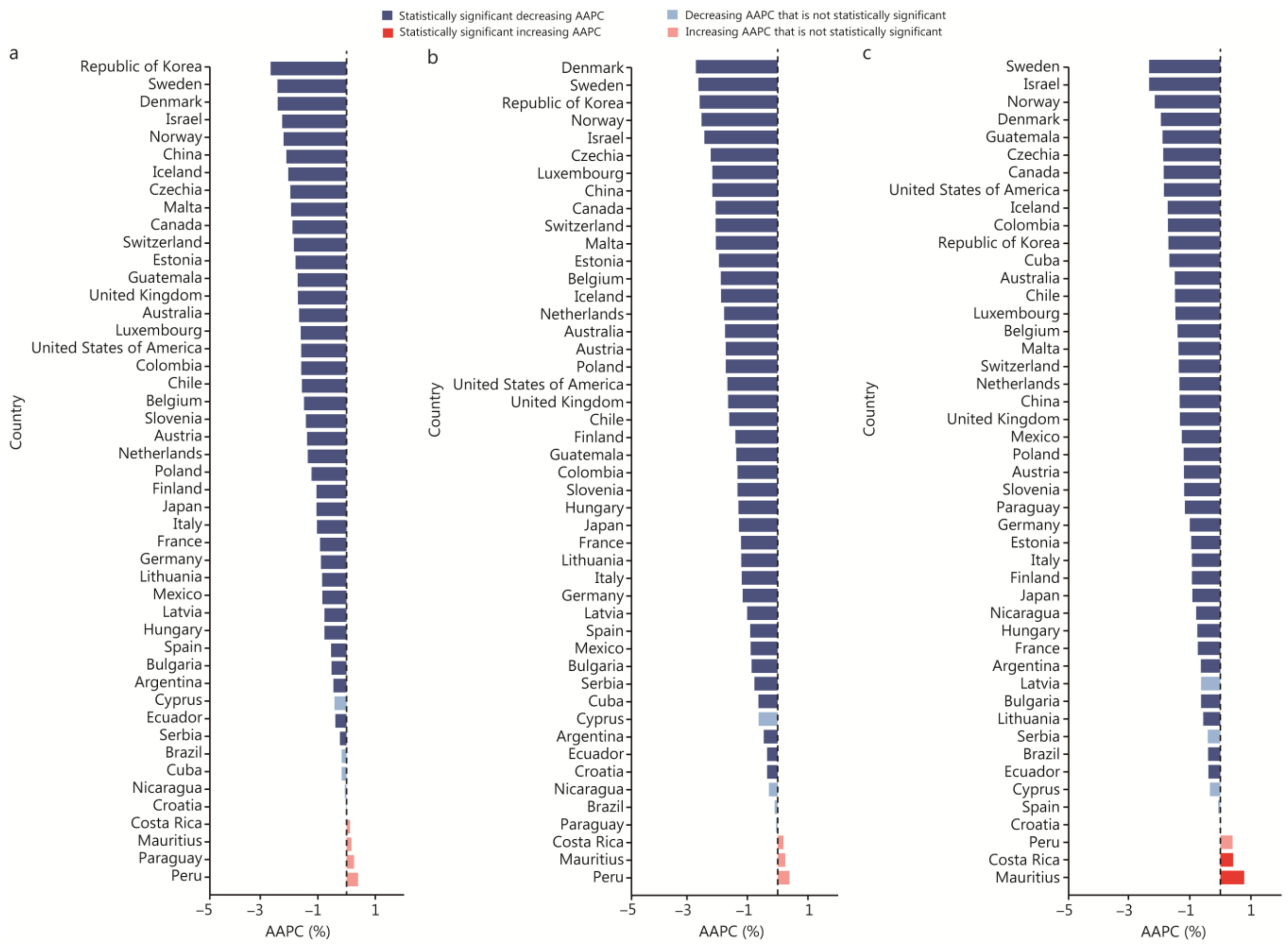

Fig. S2 AAPCs for premature cancer death indicators by country among women, 2003 − 2022. **a** Probability of premature cancer-related death. **b** The ASR of YLLs caused by cancer-related premature death. **c** The PGLEs resulting from the elimination of premature cancer death. AAPC average annual percent change, ASR age-standardized rate, YLLs years of life lost, PGLEs potential gains in life expectancy

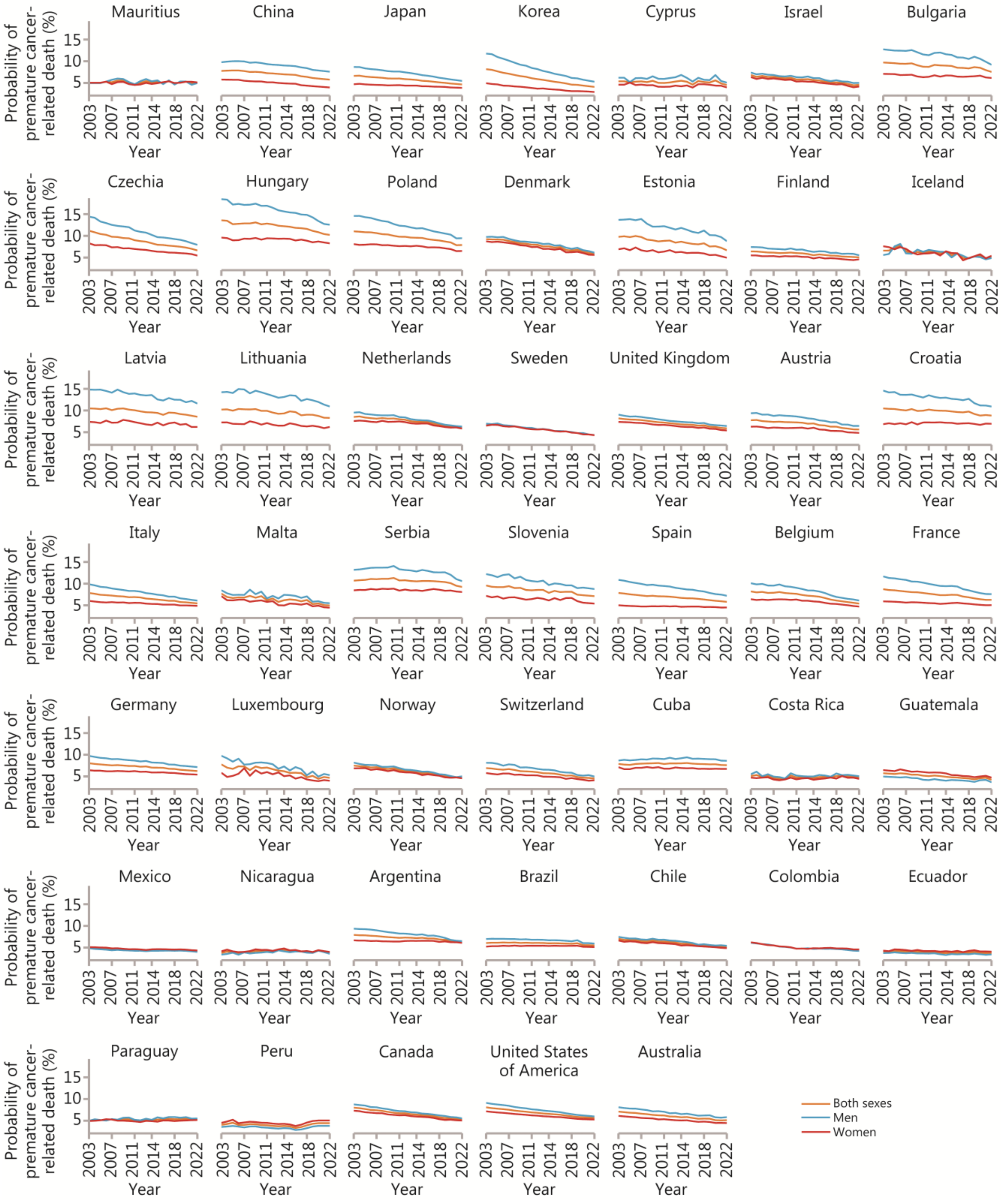

Fig. S3 The changing trends for the probability of premature cancer-related death from 2003 to 2022 by country and sex

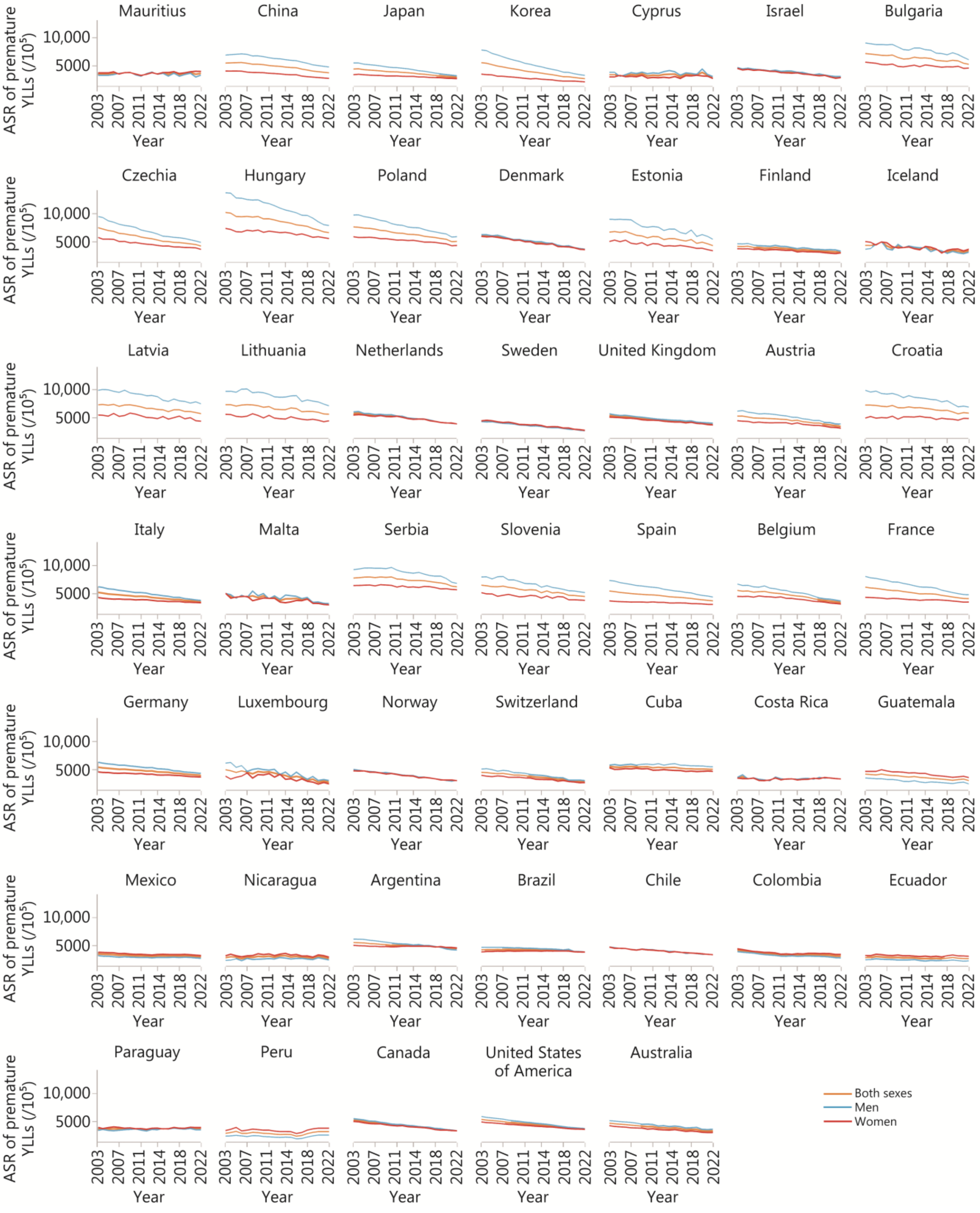

Fig. S4 The changing trends for the ASR of YLLs caused by cancer-related premature death from 2003 to 2022 by country and sex. ASR age-standardized rate, YLLs years of life lost

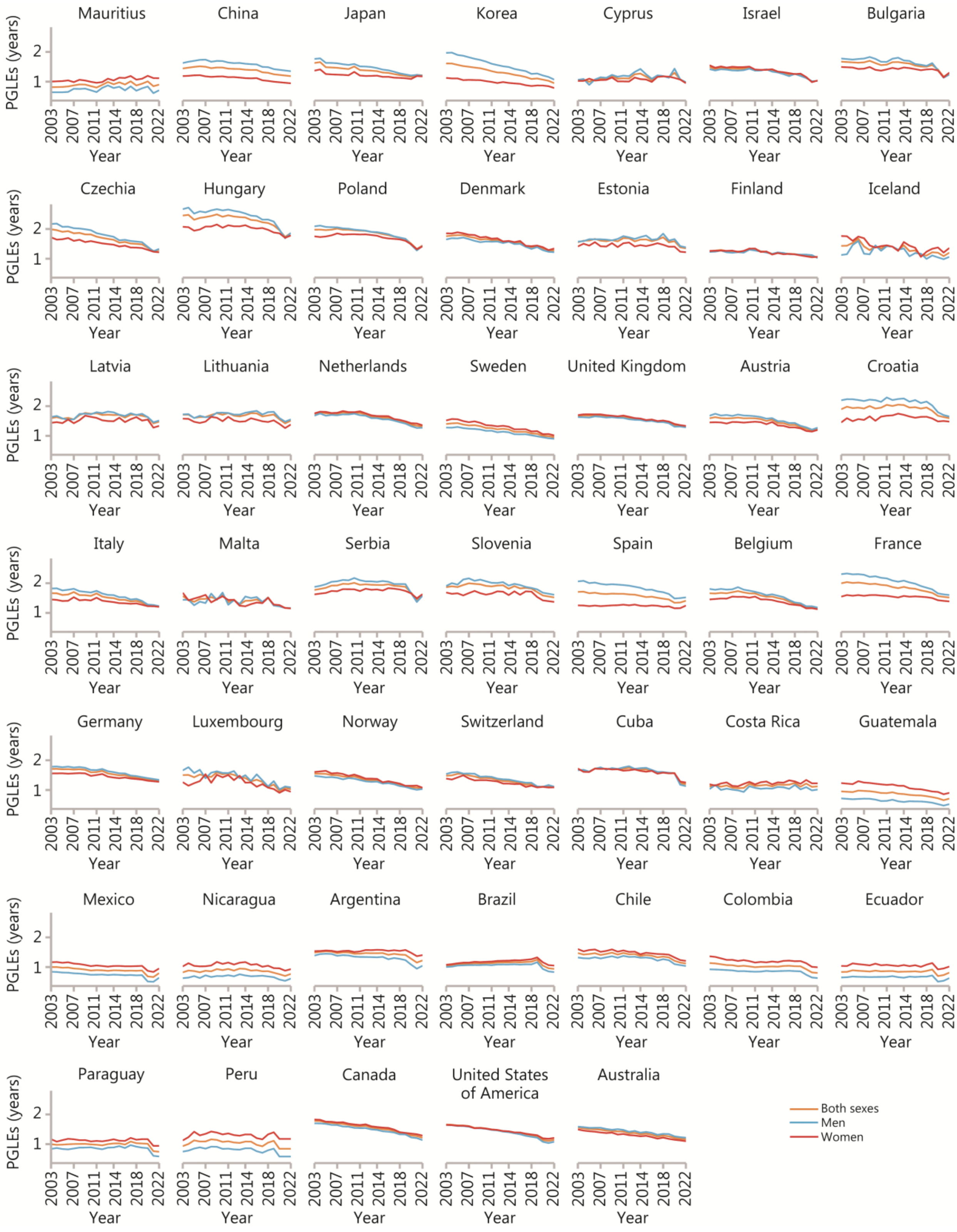

Fig. S5 The changing trends for the PGLEs resulting from the elimination of premature cancer death from 2003 to 2022 by country and sex. PGLEs potential gains in life expectancy
